# Ocular related emergencies in Spain during the COVID-19 pandemic, a multicenter study

**DOI:** 10.1186/s12886-021-02169-x

**Published:** 2021-11-27

**Authors:** Martín Puzo, Jorge Sánchez-Monroy, Carmen A. Porcar-Plana, Francisco de Asís Bartol-Puyal, Marina Dotti-Boada, Pilar Peña-Urbina, Jordi Izquierdo-Serra, Ana López-Montero, Pilar Pérez-García, Daniel Bordonaba-Bosque, Luis E. Pablo, Pilar Calvo

**Affiliations:** 1 Ophthalmology Department, Miguel Servet University Hospital, Paseo Isabel la Católica 1-3, 50009 Zaragoza, Spain; 2 Miguel Servet Ophthalmology Research Group (GIMSO), Aragón Institute for Health Research (IIS-Aragón), Zaragoza, Spain; 3 Ophthalmology Department, Hospital Virgen de los Lirios, Alcoy, Spain; 4 University of Zaragoza, Zaragoza, Spain; 5 Ophthalmology Department, Hospital Clinic, Barcelona, Spain; 6 Ophthalmology Department, Hospital Clínico San Carlos, Madrid, Spain; 7 Ophthalmology Department, Hospital Clínico Universitario, Valencia, Spain; 8 Instituto Aragonés de Ciencias de la Salud (IACS), Zaragoza, Spain

**Keywords:** COVID-19, Emergency department, Epidemiology, Ophthalmological emergencies

## Abstract

**Purpose:**

To evaluate ophthalmological emergencies (OE) during the COVID-19 pandemic comparing them with the same period of the previous year.

**Methods:**

Retrospective observational study of all OE visits in four tertiary hospitals in Spain comparing data from March 16th to April 30th, 2020 (COVID-19 period) and the same period of 2019 (pre-COVID-19 period). Severity of the conditions was assessed following *Channa et al.* publication. Data on demographics, diagnosis and treatments were collected from Electronic Medical Records.

**Results:**

During lockdown, OE significantly declined by 75.18%, from 7,730 registered in the pre-COVID-19 period to 1,928 attended during the COVID-19 period (*p *< 0.001). In 2019, 23.86% of visits were classified as emergent, 59.50% as non-emergent, and 16.65% could not be determined. In 2020, the percentage of emergent visits increased up to 29.77%, non-emergent visits significantly decreased to 52.92% (*p *< 0.001), and 17.31% of the visits were classified as “could not determine”. During the pandemic, people aged between 45 and 65 years old represented the largest attending group (37.89%), compared to 2019, where patients over 65 years were the majority (39.80%). In 2019, most frequent diagnosis was unspecified acute conjunctivitis (11.59%), followed by vitreous degeneration (6.47%), and punctate keratitis (5.86%). During the COVID-19 period, vitreous degeneration was the first cause for consultation (9.28%), followed by unspecified acute conjunctivitis (5.63%) and punctate keratitis (5.85%).

**Conclusions:**

OE visits dropped significantly during the pandemic in Spain (75.18%), although more than half were classified as non-urgent conditions, indicating a lack of understanding of the really emergent ocular pathologies among population.

**Supplementary Information:**

The online version contains supplementary material available at 10.1186/s12886-021-02169-x.

## Introduction

The SARS-CoV2 (COVID-19) pandemic forced a lifestyle change for a large part of the world’s population [1], with numerous countries declaring State of Alarm in an attempt to control the spread of the virus and avoid the collapse of health systems [2]. Spain was one of the most heavily impacted countries applying these measures, which included citizen home confinement and the reduction of work activity to essential services. However, despite these strict measures, Spain registered more than 213,435 COVID-19 infections and 24,543 related deaths in the most restrictive period of the State of Alarm (March 16th to April 31st, 2020).

Healthcare activity focused on urgent pathologies, resulting in many consultations and scheduled surgeries cancelled to avoid unnecessary face-to-face interaction. These restrictions, together with a public awareness campaign, was essential to reducing infection spread and limiting unnecessary use of emergency services to avoid overwhelming the health system.

The COVID-19 pandemic significantly impacted the practise of ophthalmology in Spanish hospitals and in other countries. Many ophthalmological societies recommended avoiding all treatments except urgent or emergent care and to limit the exposure time in the hospital in order to reduce the risk of SARS-CoV-2 transmission [3]. Consultations and surgeries were cancelled or postponed, operating theatres reassigned, and several ophthalmologists relocated to attend to patients affected by COVID-19. Data regarding the dramatic impact on different eye cares from the ophthalmological departments has been already described by the EUROCOVCATgroup [4].

Ophthalmology is a specialty with a high volume of emergencies [5, 6] and a progressive increase in ophthalmological emergencies (OE) has been noted in recent years [6, 7]. OE cover a wide range of diagnosis, with widely varying severity [6, 7]. However several national [6, 7] and international [8–10] studies suggest that almost half of emergency consultations are due to non-urgent pathologies. Although studies related to OE in Spain are scarce, the published data coincide with the international trend confirming non-emergent visits are the most frequent in the emergency department (ED), and anterior segment pathology is the most demanding cause [8]. However, it is necessary to analyze this situation during the pandemic, since as the EUROCOVCATgroup analyzed, a delay in sight/life-threatening conditions could have severe consequences and that may need immediate attention to prevent irreversible damage [4].

The purpose of this study was to evaluate the number and severity of OE presenting in four tertiary Spanish hospitals during the most restrictive period of the State of Alarm (March 16th to April 31st, 2020) and to compare them with presumed regular practice (same dates of 2019).

## Materials and methods

A retrospective, observational, multicentre study was performed, analysing the number of OE and their severity during the COVID-19 pandemic in Spain from March 16th to April 31st, 2020 and comparing them to the same period in 2019. This period corresponds to the application of the strictest containment and control measures for the coronavirus pandemic in Spain that included strict confinement at home, except for essential activities (purchase of basic goods and medical care). Only workers with essential activities were able to continue with their work activity in person [11]. All patients presenting with ophthalmic complaints to ED during the appointed period were included.

The study was performed in four tertiary hospitals in Spain: Hospital Clinic (HCB) in Barcelona, Hospital Clínico San Carlos (HCSC) in Madrid, Hospital Clínico Universitario (HCV) in Valencia, and Hospital Miguel Servet (HUMS) in Zaragoza. All hospitals included belong to the Spanish National Public Health System, provide free medical assistance to patients attending the ED and have an Ophthalmology Specialist in charge of OE 24 h a day [12].

The number and seriousness of the emergencies based on diagnosis (emergent vs. non-emergent) were the main variables studied. *Channa et al.* [8] clinical consensus was used to determine if a diagnosis was emergent, non-emergent, or it could not be determined. Some of the codes listed in our database were not present in *Channa et al.* [8] clinical consensus, therefore, they were categorized based on our own findings (eTable 1).

Demographic data and variables such as length of presentation at the hospital, the timing of the visit, treatment received and destination on discharge, were collected from the ED software and the electronic medical records.

Diagnosis represented the primary reason for the patient presenting to the ED based on the physician’s clinical judgement. Ophthalmological diagnoses were codified using the *International Classification of Diseases, Tenth Revision, Clinical Modification (ICD10-CM).* Codes for ophthalmological pathologies unrelated to traumatic injuries varied from H00 to H59 and S05 for eye traumatic injuries. Some diagnostic codes outside those parameters were added to complete the wide range of eye-related pathologies presenting to the ED (see eTable 1 in the supplement for a list of the diagnoses). If a patient had more than one diagnosis, the most emergent was selected as the primary diagnosis.

Diagnoses were distributed according to the following groups: anterior segment and ocular surface, retina, ocular inflammation, glaucoma, neuro-ophthalmology, oculoplastic and orbit, trauma, and miscellaneous (representing those diagnoses that did not fit in any other category). We did not consider for diagnosis those patients who left the ED before receiving any clinical exploration (70 patients) or those who came only for administrative purposes (298 patients). A list of the diagnosis assigned to each group is attached (eTable 1).

Age was divided into four groups (≤14 years old (yo), 15-44 yo, 45-64 yo, ≥ 65 yo). Data for the younger group may be biased as HCB referred all the children to the appropriate paediatric hospital during both pre-COVID and COVID periods. Length of presentation at the hospital was recorded within minutes of patient arrival. Timing of visits were divided into three periods: (08-16 h), (16-24 h) and (00-08 h). Variables for the received treatment were “not required”, “medical treatment”, “surgery treatment”, and “others” (including laser or intravitreal treatment). Lastly, data regarding patients’ destination on discharge were collected as: “discharge with citation”, “discharge without citation”, “hospital admission” or “other” (meaning voluntary discharge or unreported abandonment of the ED).

The design of the study followed the tenets of the Declaration of Helsinki for biomedical research and the study protocol was approved by the local ethics committee (CEICA, Zaragoza, Spain). Due to the retrospective nature of data collection, the local institutional review board committee exempted informed consent.

### Statistical analysis

All data were collected and encoded by every hospital, and statistical analysis was performed using R statistical package (A language and environment for statistical computing. R Foundation for Statistical Computing, Vienna, Austria) and Jamovi (Version 1.2) [Computer Software].

Descriptive statistics: continuous variables were described using mean and standard deviation (SD), and qualitative or categorical variables were presented through frequencies and percentages in every category.

Quantitative variables were explored with the goodness-of-fit test to a normal distribution (Shapiro-Wilk).

Bivariate analysis: the association between variables was investigated using hypothesis testing, with the comparison of proportions when the factors are qualitative variables (chi-square, Fisher’s exact test) and comparisons of means when one of them was quantitative (Student’s t-test, ANOVA), and if they do not follow normal distribution using non-parametric tests (Mann-Whitney U test or Kruskall-Wallis test). The significance level was taken to be *p* <0.05.

## Results

**Fig. 1:**
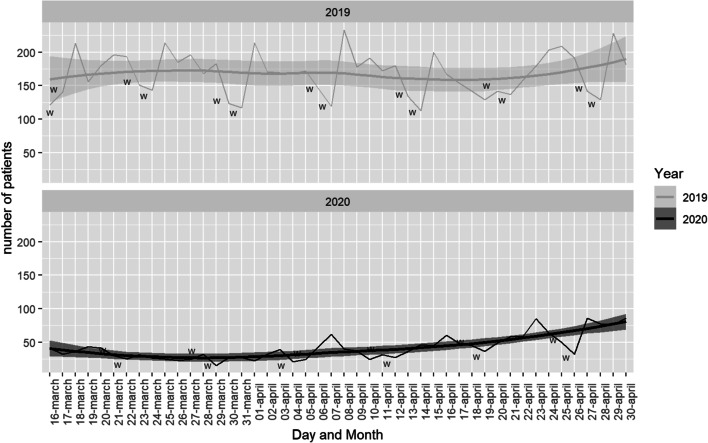
Number of visits per day during both periods. *W: weekends*

Overall, 9648 visits for OE were recorded from March 16th to April 31st, 2019 and 2020. During pre-COVID-19 period, 7730 emergencies were registered among all centres, decreasing a 75.18% in COVID-19 period, when only 1928 emergencies were attended (*p *< 0.001). In 2019, the highest number of emergencies were observed in the Monday-Friday period with a maximum peak of 231 emergencies on April 8, 2019. During the same period in 2020, the maximum was reached on April 23 with 77 emergencies. Figure 1 represents the distribution of visits per day during both years.

**Fig. 2:**
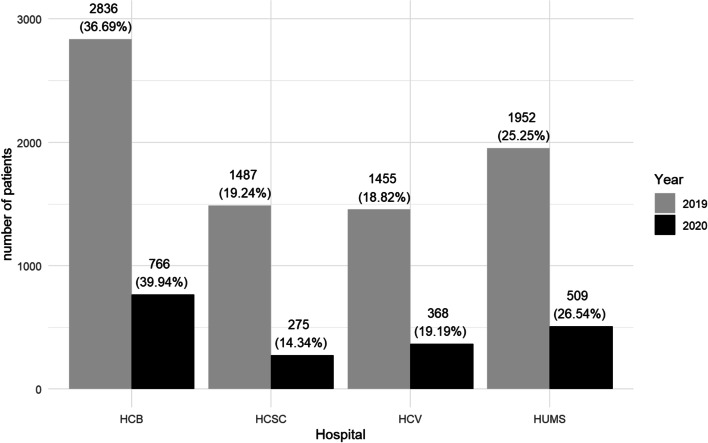
Comparison between the number of visits in each hospital per year. *HCB: Hospital Clinic Barcelona; HCSC: Hospital Clínico San Carlos; HCV: Hospital Clínico Valencia; HUMS: Hospital Universitario Miguel Servet*

The incidence of COVID was different in the regions of the 4 hospitals studied, having a more drastic impact on Madrid and Barcelona where the incidence of cases reached its peak on March 20 with (3213 and 1618 diagnosed cases respectively). While in Valencia and Zaragoza the positive cases were not so high with a maximum of 314 and 213 cases respectively [13]. The number of OE in every hospital during the time studied is displayed in Fig. 2.

**Fig. 3:**
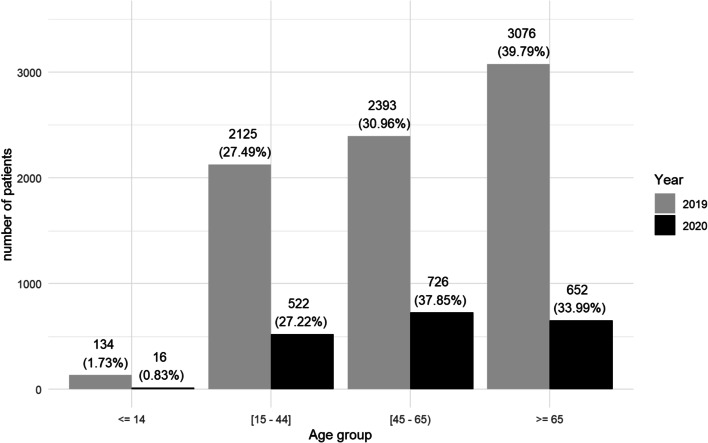
Comparison between age groups in both periods

Age distribution is displayed in Fig. 3. In 2019, the mean (SD) age at presentation was 56.42 (19.95) and patients over 65 years represented the largest group (39.80%) consulting with OE. In 2020, the mean (SD) age slightly decreased to 55.54 years (18.27) and the largest group attending to ED was between 45 and 65 years of age (37.89%). Age at presentation decreased in all the hospitals studied, however HCSC in Madrid was the one that did so in a higher range, from 57.91 (20.30) in 2019 to 53.82 (16.97) in 2020 (*p *< 0.001).

Medical assistance time was reduced significantly (*p *< 0.001) during the pandemic from a mean of (SD) 77.44 (50.23) minutes in 2019 to 62.74 (45.46) minutes during COVID-19 period. This reduction was statistically significant in all hospitals (*p *< 0.001) except in the Barcelona HCB (*p *= 0.225).

**Fig. 4:**
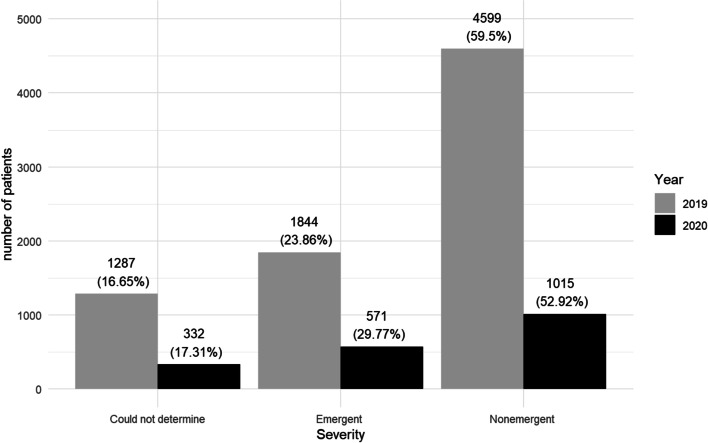
Comparison between the severity of the OE in both periods

Distribution of visits attending to their severity is displayed in Fig. 4. During COVID-19 period we observed a significant change in all the groups (*p *< 0.001). The percentage of emergent visits increased up to 29.77%, non-emergent visits decreased to 52.92%, and 17.31% of the visits were classified as “could not determine”. An increase in emerging pathology was observed in all hospitals; 6.72% in HCB, 6.71% in HUMS, 4.32% in HCV, and 4.24% in HCSC as well as a reduction in non-emergent pathology; 8.57% in HCB, 6.64% in HUMS, 5.15% in HCSC, and 2.34% in HCV.

The percentage of visits regarding diagnosis groups changed between both periods (*p *< 0.001). The main diagnosis group both in 2019 and 2020 was anterior segment and ocular surface (53.74%; 41.19%), followed by oculoplastics and orbit (14.46% ; 14.72%), retina (12.18% ; 18.87%), trauma (8.90% ; 8.09%), ocular inflammation (4.28% ; 7.28%), miscellany (2.98% ; 4.69%), neuro-ophthalmology (1.95% ; 3.50%) and glaucoma (1.51% ; 1.67%). Diagnostic groups were similar between hospitals, with anterior segment and ocular surface being the dominant one in all hospitals during 2019 and 2020, although decreasing in all of them in the time of the pandemic. An increase in retinal pathology was also seen in all of them.

ICD-10 diagnostics classified by year can be found in supplement 2 (eTable 2). Before pandemic, the most frequent diagnosis was unspecified acute conjunctivitis (H10.30) with 896 visits (11.59%), followed by vitreous degeneration (H43.819) with 500 visits (6.47%), and punctate keratitis with 453 visits (5.86%). Some diagnostic changes occurred during COVID-19 period, with vitreous degeneration being the first cause for consultation with 178 visits (9.28%) followed by unspecified acute conjunctivitis with 108 visits (5.63%) and punctate keratitis with 93 (5.85%).

No differences (*p *= 0.134) were observed in the timing pattern of the emergencies between 2019 and 2020. The busiest period occurred between 16:00 to 00:00 h, with 63.31% and 61.26% visits, respectively. This was followed by the next busy period between 08:00 to 16:00 h, with 34.85% and 37.17% respectively, and the period between 00:00 to 08:00 h with 1.84% presenting in 2019 and 1.56% presenting in 2020. There were no differences between hospitals in this matter.

**Fig. 5:**
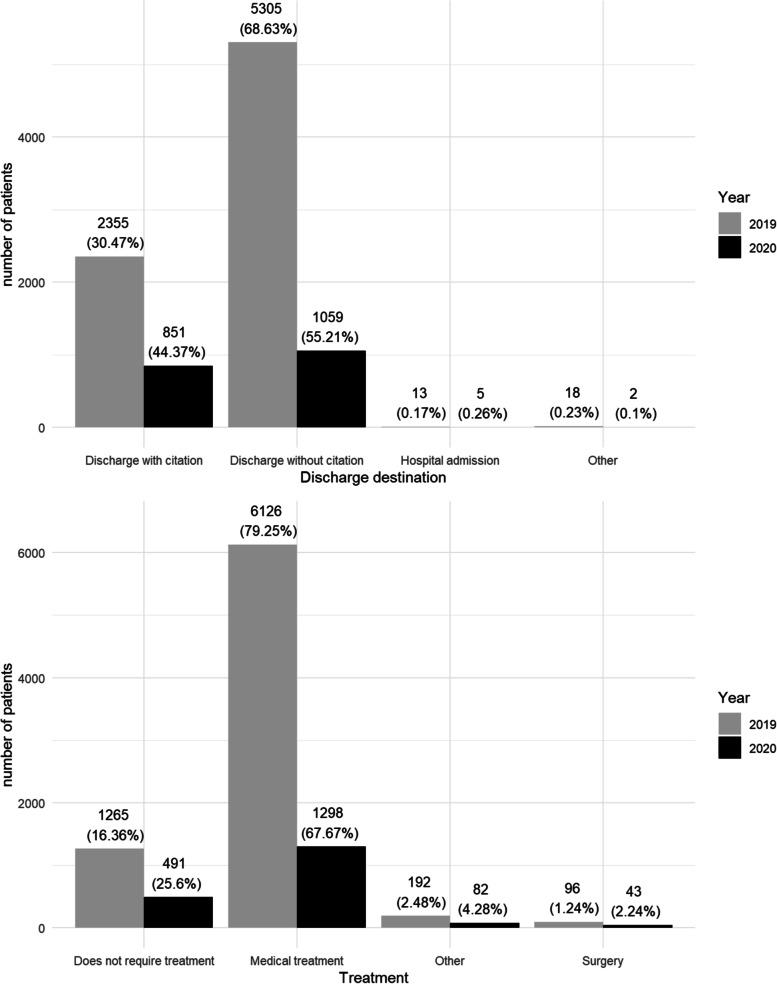
Destination at discharge and treatment received in both periods

Regarding the treatment received and the destination at discharge, we obtained statistically significant differences between both periods (*p* <0.001). Both variables are represented in Fig. 5.

## Discussion

Studying the changes in OE during this period is necessary to plan for specific strategies for future pandemic-like events and to manage health resources adequately.

During the application of the strictest measures of confinement due to the COVID-19 pandemic in Spain, the number of emergencies was significantly reduced (*p *< 0.001) compared to the same period of the previous year (a reduction of 75.18%). This reduction in the number of OE coincides with a publication that studied the number of visits to the ED globally during a similar period in Norway where the reduction of visits varied from 21 to 39% depending on the studied week [14], and with other publications that described the effects of the pandemic on OE in specific hospitals where the reduction of visits decreased more than 50% [15–17]. Fear of going to a hospital and exposure to a possible risk of infection appeared to be a critical factor in this decline as other publications suggests [16]. In accordance with our hypothesis, emergent visits increased by 5.91% and non-emergent visits decreased by 6.58%, however, the decline of the non-emergent visits were not as significant as expected. A decrease of less than 10 per cent, although significant (*p *< 0.001), indicates that patients with non-emergent conditions do not understand the severity of their pathology, and continue to present at ED. Also, this exposes them to a greater risk of SARS-Cov-2 contagion, which can be much more devastating than their eye pathology. These results agree with those of *Pellegrini et al.* [15], which highlighted a decrease of 8.7% of non-emergent conditions in 2020, and an increase of 7% of emergent OE. However, in another of the studies the increase in emergent visits was higher, reaching 11% [16].

Cities where the incidence of SARS-CoV-2 was lower [18], proportion of emergencies were higher, for example, HUMS (26.5%) in Zaragoza and HCV (18.8%) in Valencia. In Madrid, where COVID-19 dramatically affected, OE in HCSC decreased from 19.2 to 14.3%. Despite the impact of COVID-19 in Barcelona, HCB was the hospital with the highest number of patients in both 2019 and 2020. Analysing this distribution is difficult since the distribution of health resources depends on every region in Spain. This may lead to a greater dependence on hospital services in some areas during the COVID-19 period, with the partial closure of primary care centres that were primarily used to care for infected patients.

Moreover, Spain has a public, free, and universal health system in every region, where anyone can present to the ED free of charge. That translates in a greater demand of the ED, as greater health insurance coverage increases the rates of ED use as other studies have demonstrated [19]. It is important to highlight that the restrictions imposed by the Spanish central government were similar for the 4 regions studied, so the differences found are related to the population’s perception of risk, and not to the laws imposed.

The day with the fewest visits to ED in Spain was March 16th, 2020, on which the State of Alarm was decreed. As the confinement measurements became relaxed, visits increased (Fig. 1). During the pandemic, the number of daily emergencies remained homogeneous without abrupt changes between days of the week. In 2019, the number of OE were higher from Monday to Friday, and decreased over the weekend, a pattern already observed in other previous studies [7]. The application of home confinement measurements, the greater flexibility of teleworking and the suppression of leisure activities may have been able to influence the disappearance of this previous pattern (Fig. 1).

Age demographics match the national and international tendency in overall ED visits [6, 8, 9]. During the pandemic, we observed a change in the type of patient presenting to the ED. There was a significant decrease (*p *< 0.001) in the number of patients older than 65 years of age (from 39.80% to 2019 to 34.03% in 2020). The majority group during COVID-19 period was aged between 45 and 65, representing 37.89% of the visits. This could be explained because the elderly population were the most vulnerable to COVID-19 and also the age group on which the awareness campaign and health measures had the greatest impact (restriction on visits to hospital or nursing homes without opening to the public). Regarding sex of the patients, women presented more than men in both periods, in agreement with other studies [6, 8].

Pathologies were classified according to the ICD-10-CM and grouped by subspecialty. In both periods, the anterior segment and ocular surface diseases grouped the highest number of visits, followed by oculoplastic and orbit in 2019 and by retinal pathology during COVID-19 period. During the pandemic, an increase in retinal pathologies, stabilization of oculoplastic and orbital pathology, and a significant decrease (*p *< 0.001) in anterior segment and ocular surface pathologies were observed in all the involved hospitals. This may be explained as the last group includes very frequent non-urgent pathology (conjunctivitis, conjunctival haemorrhages) that do not require urgent care. Also in this group, a decrease in the incidence of acute corneal abnormalities was observed (327 corneal ulcers in 2019 versus 67 in 2020 or 118 corneal abscesses in 2019 versus 31 in 2020). This is especially relevant given the difficulty of surgical performance during the confinement period in which donations and corneal transplants were severely affected [20]. On the other hand, the increase in retinal pathology (from 12.18 to 18.87%; *p *< 0.001) is predominantly due to the relative percentage increase in vitreous pathology (6.47% in 2019, 9.18% in 2020). Also, vitreous haemorrhage (0.36% in 2019, 1.25% in 2020), macular pathology like exudative age-related macular degeneration (0.40% in 2019, 0.83% in 2020), and unspecified retinal break (from 0.43 to 0.94%) increased the relative percentage of retinal consultations. However, all the above decreased in total numbers comparing 2019 to 2020. We can assume that visual pathology (more linked to disorders related to the posterior pole) produces greater uncertainty in patients and require specialized examination to determine if these pathologies are emergent (retinal detachment) or not (myodesopsia). This linked to the saturation of primary care centres during the pandemic, and added to lack of specific exploration instruments, caused referrals more frequently than the rest of ocular pathology. These data agrees with those of previous publications both national and international [7–9, 21, 22].

In both 2019 and 2020, the vast majority of OE were classified as non-emergent. Numerous articles have analysed the significant increase in the number of visits to the ED for ocular reasons, and several have agreed that a large part of the visits are due to non-emergent pathologies [7–10] Galindo-Ferreiro et al. studied OE for a 5-year period in a Spanish hospital. They described growth in the number of OE presented year after year, from 157.34 to 10,000 inhabitants in 2013, to 162.84 per 10,000 in 2017. They also described the severity of the visits, reporting only 7.6% of non-emergent visits, although the method chosen to detect emergent or non-emergent visits differs from ours. The method chosen in our study to establish the severity of the emergencies allows us to make an in-depth comparison with the study by *Channa et al.* [9]. In this study, the number of emerging OE was 41.2%, and that of non-emerging OE was 44.3%. In our case, both periods accumulated more than 50% of non-emergent emergencies. This demonstrates that most of the OE in Spain should be better attended using non-emergent services. What produces a greater surprise is the number of non-emergent emergencies in both 2019 and 2020 in the two larger Spanish cities (Madrid and Barcelona), in which more than 60% of OE were non-emergent. We believe that this may be due to the lower use of primary care in large cities, where a large part of the population uses hospitals as the only health centre.

There is also a sizeable decrease in the total number of emerging visits between both periods, with 1,844 in 2019 (23.86%) and 571 (29.77%) in 2020. We can assume that several emerging pathologies did not occur in 2020 as a result of limitations on extra-curricular activities because of the confinement and the restrictions imposed on non-essential work. These findings correlate with patients presenting with traumatic eye injuries, decreasing (*p *< 0.001) from 660 patients in 2019 to 150 in 2020, a reduction of 510 patients. However, compared to 2019, nearly 800 patients were left unaccounted for as their diagnosis was unrelated to traumatic eye injuries and were classified as emergent, although they did not attend the ED.

The increase in severity of the OE during the COVID-19 pandemic (*p *< 0.001) saw a rise in the use of invasive treatments such as surgical treatment, laser, and intravitreal injections. This data would reflect that patients during the confinement period required more specialized treatments and attention provided by an ophthalmologist. We can also appreciate this fact in patients discharge destination; as in 2020, the number of patients discharged without a subsequent appointment lowered, and the number of patients hospitalized or discharged with citation increased.

It should also be noted that not only patients are at risk of becoming infected with SARS-CoV-2 during their visit to the ED. Since certain ophthalmological examinations require close contact with patients (e.g., slit-lamp examinations), the possibility of SARS-CoV-2 transmission during these examinations cannot be ignored. A logical finding published by The Lancet, showed that front line healthcare workers were most at risk of contracting COVID-19. Of these medical professionals, the three subspecialties at highest risk were anesthesiologists, emergency medicine physicians and ophthalmologists [23, 24].

Another consequence of the pandemic is the lack of training in ophthalmology trainees, reducing medical training and especially surgical training [25]. The current impact of COVID-19 pandemic was described as “severe” by most trainees (55.2%) in an online survey distributed in multiple countries [25], and almost 90% of the spanish ophthalmology trainees assured that the pandemic had had a negative impact on its formation [26].

This study has some limitations: its retrospective nature, subjective doctor’s diagnosis and data from only four hospitals were collected. Furthermore, it is unknown what percentage of patients who presented to ED were affected by the coronavirus disease. It would also have been interesting to analyse the visual results of the patients seen in the emergency department, as well as to compare the data from ophthalmological emergencies with the rest of hospital emergencies. A study directly aimed at the main emerging pathologies would be interesting to see how the measures have affected these types of diseases. Unfortunately, it has not been possible to conduct these investigations with the available data. Future research is necessary in this regard. However, our study is the only study published in which data is obtained from different centres, and which obtains a true picture of the characteristics of ophthalmological emergencies during the pandemic.

In conclusion, during the coronavirus pandemic, OE in Spain decreased by more than 75% compared to the previous year, and slightly increased in severity. However, more than half of the patients who requested medical assistance did not have emergent pathologies. Acute ophthalmic conditions can be difficult to manage for non-ophthalmologists or nurses because they can compromise vision without obvious clinical findings. Using the same classification system for general acute medical conditions and ophthalmic pathology may not be the best option to discriminate the severity of ophthalmic conditions. Having a specific triage system for ophthalmological emergencies would better recognize the severity of the different ophthalmological conditions, improve the efficiency of the emergency system and reduce waiting times [6, 27], which is more relevant than ever in exceptional situations such as this pandemic. Also, self-triage systems [28] and other computer systems [29] have been described for the classification of patients with ocular pathologies, whose development and standardization could optimize emergency screening in our specialty. These tools are especially useful in the pandemic because they eliminate the need to go to health centres and reduces the waiting time for those who do need urgent attention. Some of them are already being used in retinal pathologies in which time plays a critical role in the evolution of the disease [30, 31].

Finally, it is essential to highlight again the need for an eye emergency system that allows rapid, efficient, and decisive care for patients with real emergent conditions. Evaluation of electronic medical records are highly important to be prepared for this kind of situation and for creating a good strategy such as telemedicine. Sharing what we have been through during this unpredictable situation and sharing these data will make us ready for the future one.

## Supplementary Information

**Additional file 1:** **ETable1.** Diagnosis codes classified according to severity and diagnostic group.

**Additional file 2:** **ETable2. **Incidence of diagnosis per year.

## Data Availability

The datasets used and/or analysed during the current study are available from the corresponding author on reasonable request.

## Abbreviations

OE: Ophthalmological emergencies
ED: Emergency department
HCB: Hospital Clinic of Barcelona
HCSC: Hospital Clínico San Carlos
HCV: Hospital Clínico Of Valencia
HUMS: Hospital Universitario Miguel Servet
