# Supplementary material for: Ocular related emergencies in Spain during the COVID-19 pandemic, a multicenter study
Source: BMC Ophthalmol. 2021 Nov 27;21:408. doi: 10.1186/s12886-021-02169-x (PMC8626758; doi:10.1186/s12886-021-02169-x)
Supplement: Supplementary file 1 — Additional file 1: ETable1. Diagnosis codes classified according to severity and diagnostic group. [file 12886_2021_2169_MOESM1_ESM.docx]

| **ETable1. Diagnosis codes classified according to severity and diagnostic group** | | | |
| --- | --- | --- | --- |
| **ICD‐10 Diagnosis Code** | **ICD-10 Diagnosis** | **Likely  Emergent?** | **Diagnosis group** |
| B00.50 | Herpesviral ocular disease | Could not  determine | Anterior pole / Ocular surface |
| B00.52 | Herpesviral keratitis | Yes | Anterior pole / Ocular surface |
| E08.32 | Diabetes mellitus due to underlying condition with mild nonproliferative diabetic retinopathy | No | Retina |
| E08.34 | Diabetes mellitus due to underlying condition with severe nonproliferative diabetic retinopathy | No | Retina |
| E11.32 | Type 2 diabetes mellitus with mild nonproliferative diabetic retinopathy | No | Retina |
| E13.31 | Other specified diabetes mellitus with unspecified diabetic retinopathy | No | Retina |
| E13.35 | Other specified diabetes mellitus with proliferative diabetic retinopathy | No | Retina |
| G43.1 | Migraine with aura | Could not  determine | Neuroophthalmology |
| H00.01 | Hordeolum externum | No | Oculoplastics / Orbit |
| H00.023 | Hordeolum internum right eye, unspecified eyelid | No | Oculoplastics / Orbit |
| H00.03 | Abscess of eyelid | Yes | Oculoplastics / Orbit |
| H00.19 | Chalazion unspecified eye, unspecified eyelid | No | Oculoplastics / Orbit |
| H01.009 | Unspecified blepharitis unspecified eye, unspecified eyelid | No | Oculoplastics / Orbit |
| H01.019 | Ulcerative blepharitis unspecified eye, unspecified eyelid | No | Oculoplastics / Orbit |
| H01.029 | Squamous blepharitis unspecified eye, unspecified eyelid | No | Oculoplastics / Orbit |
| H01.119 | Allergic dermatitis of unspecified eye, unspecified eyelid | No | Oculoplastics / Orbit |
| H01.129 | Discoid lupus erythematosus of unspecified eye, unspecified eyelid | No | Oculoplastics / Orbit |
| H01.139 | Eczematous dermatitis of unspecified eye, unspecified eyelid | No | Oculoplastics / Orbit |
| H01.149 | Xeroderma of unspecified eye, unspecified eyelid | No | Oculoplastics / Orbit |
| H01.8 | Other specified inflammations of eyelid | No | Oculoplastics / Orbit |
| H01.8 | Other specified inflammations of eyelid | No | Oculoplastics / Orbit |
| H01.8 | Other specified inflammations of eyelid | No | Oculoplastics / Orbit |
| H01.9 | Unspecified inflammation of eyelid | No | Oculoplastics / Orbit |
| H02.009 | Unspecified entropion of unspecified eye, unspecified eyelid | No | Oculoplastics / Orbit |
| H02.019 | Cicatricial entropion of unspecified eye, unspecified eyelid | No | Oculoplastics / Orbit |
| H02.033 | Senile entropion of right eye, unspecified eyelid | No | Oculoplastics / Orbit |
| H02.049 | Spastic entropion of unspecified eye, unspecified eyelid | No | Oculoplastics / Orbit |
| H02.059 | Trichiasis without entropion unspecified eye, unspecified eyelid | No | Oculoplastics / Orbit |
| **ICD‐10 Diagnosis Code** | **ICD-10 Diagnosis** | **Likely  Emergent?** | **Diagnosis group** |
| H02.109 | Unspecified ectropion of unspecified eye, unspecified eyelid | No | Oculoplastics / Orbit |
| H02.119 | Cicatricial ectropion of unspecified eye, unspecified eyelid | No | Oculoplastics / Orbit |
| H02.129 | Mechanical ectropion of unspecified eye, unspecified eyelid | No | Oculoplastics / Orbit |
| H02.139 | Senile ectropion of unspecified eye, unspecified eyelid | No | Oculoplastics / Orbit |
| H02.149 | Spastic ectropion of unspecified eye, unspecified eyelid | No | Oculoplastics / Orbit |
| H02.209 | Unspecified lagophthalmos unspecified eye, unspecified eyelid | No | Oculoplastics / Orbit |
| H02.219 | Cicatricial lagophthalmos unspecified eye, unspecified eyelid | No | Oculoplastics / Orbit |
| H02.239 | Paralytic lagophthalmos unspecified eye, unspecified eyelid | Could not  determine | Oculoplastics / Orbit |
| H02.30 | Blepharochalasis unspecified eye, unspecified eyelid | No | Oculoplastics / Orbit |
| H02.409 | Unspecified ptosis of unspecified eyelid | Could not determine | Oculoplastics / Orbit |
| H02.419 | Mechanical ptosis of unspecified eyelid | No | Oculoplastics / Orbit |
| H02.429 | Myogenic ptosis of unspecified eyelid | Could not  determine | Oculoplastics / Orbit |
| H02.439 | Paralytic ptosis unspecified eyelid | Yes | Oculoplastics / Orbit |
| H02.519 | Abnormal innervation syndrome unspecified eye, unspecified eyelid | No | Oculoplastics / Orbit |
| H02.529 | Blepharophimosis unspecified eye, unspecified lid | No | Oculoplastics / Orbit |
| H02.539 | Eyelid retraction unspecified eye, unspecified lid | No | Oculoplastics / Orbit |
| H02.59 | Other disorders affecting eyelid function | Could not determine | Oculoplastics / Orbit |
| H02.59 | Other disorders affecting eyelid function | Could not determine | Oculoplastics / Orbit |
| H02.60 | Xanthelasma of unspecified eye, unspecified eyelid | No | Oculoplastics / Orbit |
| H02.70 | Unspecified degenerative disorders of eyelid and periocular area | No | Oculoplastics / Orbit |
| H02.719 | Chloasma of unspecified eye, unspecified eyelid and periocular area | No | Oculoplastics / Orbit |
| H02.729 | Madarosis of unspecified eye, unspecified eyelid and periocular area | No | Oculoplastics / Orbit |
| H02.739 | Vitiligo of unspecified eye, unspecified eyelid and periocular area | No | Oculoplastics / Orbit |
| H02.79 | Other degenerative disorders of eyelid and periocular area | No | Oculoplastics / Orbit |
| H02.819 | Retained foreign body in unspecified eye, unspecified eyelid | Yes | Oculoplastics / Orbit |
| H02.829 | Cysts of unspecified eye, unspecified eyelid | No | Oculoplastics / Orbit |
| H02.839 | Dermatochalasis of unspecified eye, unspecified eyelid | No | Oculoplastics / Orbit |
| H02.849 | Edema of unspecified eye, unspecified eyelid | No | Oculoplastics / Orbit |
| **ICD‐10 Diagnosis Code** | **ICD-10 Diagnosis** | **Likely  Emergent?** | **Diagnosis group** |
| H02.859 | Elephantiasis of unspecified eye, unspecified eyelid | No | Oculoplastics / Orbit |
| H02.873 | Vascular anomalies of right eye, unspecified eyelid | No | Oculoplastics / Orbit |
| H02.89 | Other specified disorders of eyelid | No | Oculoplastics / Orbit |
| H02.89 | Other specified disorders of eyelid | Yes | Oculoplastics / Orbit |
| H02.9 | Unspecified disorder of eyelid | No | Oculoplastics / Orbit |
| H04.009 | Unspecified dacryoadenitis, unspecified lacrimal gland | Could not  determine | Oculoplastics / Orbit |
| H04.019 | Acute dacryoadenitis, unspecified lacrimal gland | Yes | Oculoplastics / Orbit |
| H04.029 | Chronic dacryoadenitis, unspecified lacrimal gland | No | Oculoplastics / Orbit |
| H04.039 | Chronic enlargement of unspecified lacrimal gland | No | Oculoplastics / Orbit |
| H04.119 | Dacryops of unspecified lacrimal gland | No | Oculoplastics / Orbit |
| H04.129 | Dry eye syndrome of unspecified lacrimal gland | No | Oculoplastics / Orbit |
| H04.139 | Lacrimal cyst, unspecified lacrimal gland | No | Oculoplastics / Orbit |
| H04.149 | Primary lacrimal gland atrophy, unspecified lacrimal gland | No | Oculoplastics / Orbit |
| H04.159 | Secondary lacrimal gland atrophy, unspecified lacrimal gland | No | Oculoplastics / Orbit |
| H04.169 | Lacrimal gland dislocation, unspecified lacrimal gland | Could not determine | Oculoplastics / Orbit |
| H04.209 | Unspecified epiphora, unspecified side | No | Oculoplastics / Orbit |
| H04.219 | Epiphora due to excess lacrimation, unspecified lacrimal gland | No | Oculoplastics / Orbit |
| H04.229 | Epiphora due to insufficient drainage, unspecified side | No | Oculoplastics / Orbit |
| H04.309 | Unspecified dacryocystitis of unspecified lacrimal passage | Could not determine | Oculoplastics / Orbit |
| H04.319 | Phlegmonous dacryocystitis of unspecified lacrimal passage | Could not determine | Oculoplastics / Orbit |
| H04.329 | Acute dacryocystitis of unspecified lacrimal passage | Yes | Oculoplastics / Orbit |
| H04.339 | Acute lacrimal canaliculitis of unspecified lacrimal passage | Yes | Oculoplastics / Orbit |
| H04.419 | Chronic dacryocystitis of unspecified lacrimal passage | No | Oculoplastics / Orbit |
| H04.429 | Chronic lacrimal canaliculitis of unspecified lacrimal passage | No | Oculoplastics / Orbit |
| H04.439 | Chronic lacrimal mucocele of unspecified lacrimal passage | No | Oculoplastics / Orbit |
| H04.519 | Dacryolith of unspecified lacrimal passage | No | Oculoplastics / Orbit |
| H04.529 | Eversion of unspecified lacrimal punctum | No | Oculoplastics / Orbit |
| H04.539 | Neonatal obstruction of unspecified nasolacrimal duct | No | Oculoplastics / Orbit |
| H04.549 | Stenosis of unspecified lacrimal canaliculi | No | Oculoplastics / Orbit |
| **ICD‐10 Diagnosis Code** | **ICD-10 Diagnosis** | **Likely  Emergent?** | **Diagnosis group** |
| H04.559 | Acquired stenosis of unspecified nasolacrimal duct | No | Oculoplastics / Orbit |
| H04.569 | Stenosis of unspecified lacrimal punctum | No | Oculoplastics / Orbit |
| H04.579 | Stenosis of unspecified lacrimal sac | No | Oculoplastics / Orbit |
| H04.619 | Lacrimal fistula unspecified lacrimal passage | No | Oculoplastics / Orbit |
| H04.69 | Other changes of lacrimal passages | Could not determine | Oculoplastics / Orbit |
| H04.819 | Granuloma of unspecified lacrimal passage | No | Oculoplastics / Orbit |
| H04.89 | Other disorders of lacrimal system | Could not  determine | Oculoplastics / Orbit |
| H04.9 | Disorder of lacrimal system, unspecified | Could not determine | Oculoplastics / Orbit |
| H05.00 | Unspecified acute inflammation of orbit | Yes | Oculoplastics / Orbit |
| H05.01 | Cellulitis of ORBIT | Yes | Oculoplastics / Orbit |
| H05.029 | Osteomyelitis of unspecified orbit | Yes | Oculoplastics / Orbit |
| H05.039 | Periostitis of unspecified orbit | Yes | Oculoplastics / Orbit |
| H05.049 | Tenonitis of unspecified orbit | No | Oculoplastics / Orbit |
| H05.10 | Unspecified chronic inflammatory disorders of orbit | No | Oculoplastics / Orbit |
| H05.119 | Granuloma of unspecified orbit | Yes | Oculoplastics / Orbit |
| H05.129 | Orbital myositis, unspecified orbit | Yes | Oculoplastics / Orbit |
| H05.20 | Unspecified exophthalmos | Could not determine | Oculoplastics / Orbit |
| H05.219 | Displacement (lateral) of globe, unspecified eye | No | Oculoplastics / Orbit |
| H05.229 | Edema of unspecified orbit | Could not determine | Oculoplastics / Orbit |
| H05.239 | Hemorrhage of unspecified orbit | Yes | Oculoplastics / Orbit |
| H05.249 | Constant exophthalmos, unspecified eye | No | Oculoplastics / Orbit |
| H05.259 | Intermittent exophthalmos, unspecified eye | No | Oculoplastics / Orbit |
| H05.269 | Pulsating exophthalmos, unspecified eye | Yes | Oculoplastics / Orbit |
| H05.30 | Unspecified deformity of orbit | Could not determine | Oculoplastics / Orbit |
| H05.319 | Atrophy of unspecified orbit | No | Oculoplastics / Orbit |
| H05.339 | Deformity of unspecified orbit due to trauma or surgery | Could not determine | Oculoplastics / Orbit |
| H05.349 | Enlargement of unspecified orbit | No | Oculoplastics / Orbit |
| H05.359 | Exostosis of unspecified orbit | No | Oculoplastics / Orbit |
| H05.409 | Unspecified enophthalmos, unspecified eye | No | Oculoplastics / Orbit |
| H05.419 | Enophthalmos due to atrophy of orbital tissue, unspecified eye | No | Oculoplastics / Orbit |
| **ICD‐10 Diagnosis Code** | **ICD-10 Diagnosis** | **Likely  Emergent?** | **Diagnosis group** |
| H05.429 | Enophthalmos due to trauma or surgery, unspecified eye | No | Oculoplastics / Orbit |
| H05.50 | Retained (old) foreign body following penetrating wound of unspecified orbit | No | Oculoplastics / Orbit |
| H05.819 | Cyst of unspecified orbit | No | Oculoplastics / Orbit |
| H05.829 | Myopathy of extraocular muscles, unspecified orbit | No | Oculoplastics / Orbit |
| H05.89 | Other disorders of orbit | Could not  determine | Oculoplastics / Orbit |
| H05.89 | Other disorders of orbit | No | Oculoplastics / Orbit |
| H05.89 | Other disorders of orbit | Could not  determine | Oculoplastics / Orbit |
| H05.9 | Unspecified disorder of orbit | Could not  determine | Oculoplastics / Orbit |
| H10.019 | Acute follicular conjunctivitis, unspecified eye | No | Anterior pole / Ocular surface |
| H10.029 | Other mucopurulent conjunctivitis, unspecified eye | No | Anterior pole / Ocular surface |
| H10.10 | Acute atopic conjunctivitis, unspecified eye | No | Anterior pole / Ocular surface |
| H10.219 | Acute toxic conjunctivitis, unspecified eye | No | Anterior pole / Ocular surface |
| H10.229 | Pseudomembranous conjunctivitis, unspecified eye | No | Anterior pole / Ocular surface |
| H10.23 | Serous conjunctivitis, except viral | No | Anterior pole / Ocular surface |
| H10.30 | Unspecified acute conjunctivitis, unspecified eye | No | Anterior pole / Ocular surface |
| H10.409 | Unspecified chronic conjunctivitis, unspecified eye | No | Anterior pole / Ocular surface |
| H10.429 | Simple chronic conjunctivitis, unspecified eye | No | Anterior pole / Ocular surface |
| H10.439 | Chronic follicular conjunctivitis, unspecified eye | No | Anterior pole / Ocular surface |
| H10.44 | Vernal conjunctivitis | No | Anterior pole / Ocular surface |
| H10.45 | Other chronic allergic conjunctivitis | No | Anterior pole / Ocular surface |
| H10.509 | Unspecified blepharoconjunctivitis, unspecified eye | No | Anterior pole / Ocular surface |
| H10.529 | Angular blepharoconjunctivitis, unspecified eye | No | Anterior pole / Ocular surface |
| H10.539 | Contact blepharoconjunctivitis, unspecified eye | No | Anterior pole / Ocular surface |
| H10.819 | Pingueculitis, unspecified eye | No | Anterior pole / Ocular surface |
| H10.89 | Other conjunctivitis | No | Anterior pole / Ocular surface |
| H10.89 | Other conjunctivitis | No | Anterior pole / Ocular surface |
| H10.89 | Other conjunctivitis | No | Anterior pole / Ocular surface |
| H10.89, B60.12 | Other conjunctivitis, Conjunctivitis due to Acanthamoeba | No | Anterior pole / Ocular surface |
| **ICD‐10 Diagnosis Code** | **ICD-10 Diagnosis** | **Likely  Emergent?** | **Diagnosis group** |
| H10.9 | Unspecified conjunctivitis | No | Anterior pole / Ocular surface |
| H11.009 | Unspecified pterygium of unspecified eye | No | Anterior pole / Ocular surface |
| H11.029 | Central pterygium of unspecified eye | No | Anterior pole / Ocular surface |
| H11.039 | Double pterygium of unspecified eye | No | Anterior pole / Ocular surface |
| H11.049 | Peripheral pterygium, stationary, unspecified eye | No | Anterior pole / Ocular surface |
| H11.059 | Peripheral pterygium, progressive, unspecified eye | No | Anterior pole / Ocular surface |
| H11.069 | Recurrent pterygium of unspecified eye | No | Anterior pole / Ocular surface |
| H11.10 | Unspecified conjunctival degenerations | No | Anterior pole / Ocular surface |
| H11.119 | Conjunctival deposits, unspecified eye | No | Anterior pole / Ocular surface |
| H11.129 | Conjunctival concretions, unspecified eye | No | Anterior pole / Ocular surface |
| H11.139 | Conjunctival pigmentations, unspecified eye | No | Anterior pole / Ocular surface |
| H11.149 | Conjunctival xerosis, unspecified, unspecified eye | No | Anterior pole / Ocular surface |
| H11.159 | Pinguecula, unspecified eye | No | Anterior pole / Ocular surface |
| H11.219 | Conjunctival adhesions and strands (localized), unspecified eye | No | Anterior pole / Ocular surface |
| H11.229 | Conjunctival granuloma, unspecified | No | Anterior pole / Ocular surface |
| H11.239 | Symblepharon, unspecified eye | No | Anterior pole / Ocular surface |
| H11.249 | Scarring of conjunctiva, unspecified eye | No | Anterior pole / Ocular surface |
| H11.30 | Conjunctival hemorrhage, unspecified eye | No | Anterior pole / Ocular surface |
| H11.419 | Vascular abnormalities of conjunctiva, unspecified eye | No | Anterior pole / Ocular surface |
| H11.429 | Conjunctival edema, unspecified eye | No | Anterior pole / Ocular surface |
| H11.43 | Conjuntival Hiperhemia | Could not  determine | Anterior pole / Ocular surface |
| H11.439 | Conjunctival hyperemia, unspecified eye | No | Anterior pole / Ocular surface |
| H11.44 | Conjuntival Cysts | Could not  determine | Anterior pole / Ocular surface |
| H11.449 | Conjunctival cysts, unspecified eye | No | Anterior pole / Ocular surface |
| H11.819 | Pseudopterygium of conjunctiva, unspecified eye | No | Anterior pole / Ocular surface |
| H11.829 | Conjunctivochalasis, unspecified eye | No | Anterior pole / Ocular surface |
| H11.89 | Other specified disorders of conjunctiva | No | Anterior pole / Ocular surface |
| H11.9 | Unspecified disorder of conjunctiva | No | Anterior pole / Ocular surface |
| **ICD‐10 Diagnosis Code** | **ICD-10 Diagnosis** | **Likely  Emergent?** | **Diagnosis group** |
| H15.009 | Unspecified scleritis, unspecified eye | Could not determine | Anterior pole / Ocular surface |
| H15.019 | Anterior scleritis, unspecified eye | Yes | Anterior pole / Ocular surface |
| H15.029 | Brawny scleritis, unspecified eye | No | Anterior pole / Ocular surface |
| H15.039 | Posterior scleritis, unspecified eye | Yes | Anterior pole / Ocular surface |
| H15.049 | Scleritis with corneal involvement, unspecified eye | Yes | Anterior pole / Ocular surface |
| H15.059 | Scleromalacia perforans, unspecified eye | Yes | Anterior pole / Ocular surface |
| H15.099 | Other scleritis, unspecified eye | Yes | Anterior pole / Ocular surface |
| H15.119 | Episcleritis periodica fugax, unspecified eye | No | Anterior pole / Ocular surface |
| H15.129 | Nodular episcleritis, unspecified eye | No | Anterior pole / Ocular surface |
| H15.819 | Equatorial staphyloma, unspecified eye | No | Anterior pole / Ocular surface |
| H15.829 | Localized anterior staphyloma, unspecified eye | No | Anterior pole / Ocular surface |
| H15.839 | Staphyloma posticum, unspecified eye | No | Anterior pole / Ocular surface |
| H15.849 | Scleral ectasia, unspecified eye | No | Anterior pole / Ocular surface |
| H15.859 | Ring staphyloma, unspecified eye | No | Anterior pole / Ocular surface |
| H15.89 | Other disorders of sclera | No | Anterior pole / Ocular surface |
| H15.9 | Unspecified disorder of sclera | Could not determine | Anterior pole / Ocular surface |
| H16.009 | Unspecified corneal ulcer, unspecified eye | Yes | Anterior pole / Ocular surface |
| H16.019 | Central corneal ulcer, unspecified eye | Yes | Anterior pole / Ocular surface |
| H16.039 | Corneal ulcer with hypopyon, unspecified eye | Yes | Anterior pole / Ocular surface |
| H16.049 | Marginal corneal ulcer, unspecified eye | Yes | Anterior pole / Ocular surface |
| H16.059 | Mooren's corneal ulcer, unspecified eye | Yes | Anterior pole / Ocular surface |
| H16.069 | Mycotic corneal ulcer, unspecified eye | Yes | Anterior pole / Ocular surface |
| H16.079 | Perforated corneal ulcer, unspecified eye | Yes | Anterior pole / Ocular surface |
| H16.109 | Unspecified superficial keratitis, unspecified eye | Could not  determine | Anterior pole / Ocular surface |
| H16.119 | Macular keratitis, unspecified eye | Could not determine | Anterior pole / Ocular surface |
| H16.129 | Filamentary keratitis, unspecified eye | No | Anterior pole / Ocular surface |
| H16.139 | Photokeratitis, unspecified eye | No | Anterior pole / Ocular surface |
| H16.149 | Punctate keratitis, unspecified eye | Could not determine | Anterior pole / Ocular surface |
| **ICD‐10 Diagnosis Code** | **ICD-10 Diagnosis** | **Likely  Emergent?** | **Diagnosis group** |
| H16.209 | Unspecified keratoconjunctivitis, unspecified eye | No | Anterior pole / Ocular surface |
| H16.219 | Exposure keratoconjunctivitis, unspecified eye | No | Anterior pole / Ocular surface |
| H16.229 | Keratoconjunctivitis sicca, not specified as Sjogren's, unspecified eye | No | Anterior pole / Ocular surface |
| H16.239 | Neurotrophic keratoconjunctivitis, unspecified eye | No | Anterior pole / Ocular surface |
| H16.259 | Phlyctenular keratoconjunctivitis, unspecified eye | No | Anterior pole / Ocular surface |
| H16.269 | Vernal keratoconjunctivitis, with limbar and corneal involvement, unspecified eye | No | Anterior pole / Ocular surface |
| H16.299 | Other keratoconjunctivitis, unspecified eye | No | Anterior pole / Ocular surface |
| H16.299 | Other keratoconjunctivitis, unspecified eye | No | Anterior pole / Ocular surface |
| H16.309 | Unspecified interstitial keratitis, unspecified eye | No | Anterior pole / Ocular surface |
| H16.319 | Corneal abscess, unspecified eye | Yes | Anterior pole / Ocular surface |
| H16.329 | Diffuse interstitial keratitis, unspecified eye | No | Anterior pole / Ocular surface |
| H16.339 | Sclerosing keratitis, unspecified eye | No | Anterior pole / Ocular surface |
| H16.399, A18.52 | Other interstitial and deep keratitis, unspecified eye, Tuberculous keratitis | Could not determine | Anterior pole / Ocular surface |
| H16.429 | Pannus (corneal), unspecified eye | No | Anterior pole / Ocular surface |
| H16.439 | Localized vascularization of cornea, unspecified eye | Could not  determine | Anterior pole / Ocular surface |
| H16.8, B60.13 | Other keratitis, Keratoconjunctivitis due to Acanthamoeba | Could not determine | Anterior pole / Ocular surface |
| H16.9 | Unspecified keratitis | Could not determine | Anterior pole / Ocular surface |
| H17.10 | Central corneal opacity, unspecified eye | No | Anterior pole / Ocular surface |
| H17.819 | Minor opacity of cornea, unspecified eye | No | Anterior pole / Ocular surface |
| H17.829 | Peripheral opacity of cornea, unspecified eye | No | Anterior pole / Ocular surface |
| H17.9 | Unspecified corneal scar and opacity | Could not determine | Anterior pole / Ocular surface |
| H18.009 | Unspecified corneal deposit, unspecified eye | No | Anterior pole / Ocular surface |
| H18.019 | Anterior corneal pigmentations, unspecified eye | No | Anterior pole / Ocular surface |
| H18.039 | Corneal deposits in metabolic disorders, unspecified eye | No | Anterior pole / Ocular surface |
| H18.049 | Kayser-Fleischer ring, unspecified eye | No | Anterior pole / Ocular surface |
| H18.059 | Posterior corneal pigmentations, unspecified eye | No | Anterior pole / Ocular surface |
| H18.069 | Stromal corneal pigmentations, unspecified eye | No | Anterior pole / Ocular surface |
| H18.069 | Stromal corneal pigmentations, unspecified eye | No | Anterior pole / Ocular surface |
| **ICD‐10 Diagnosis Code** | **ICD-10 Diagnosis** | **Likely  Emergent?** | **Diagnosis group** |
| H18.10 | Bullous keratopathy, unspecified eye | No | Anterior pole / Ocular surface |
| H18.20 | Unspecified corneal edema | No | Anterior pole / Ocular surface |
| H18.219 | Corneal edema secondary to contact lens, unspecified eye | No | Anterior pole / Ocular surface |
| H18.229 | Idiopathic corneal edema, unspecified eye | No | Anterior pole / Ocular surface |
| H18.239 | Secondary corneal edema, unspecified eye | No | Anterior pole / Ocular surface |
| H18.30 | Unspecified corneal membrane change | No | Anterior pole / Ocular surface |
| H18.329 | Folds in Descemet's membrane, unspecified eye | No | Anterior pole / Ocular surface |
| H18.339 | Rupture in Descemet's membrane, unspecified eye | Yes | Anterior pole / Ocular surface |
| H18.40 | Unspecified corneal degeneration | No | Anterior pole / Ocular surface |
| H18.419 | Arcus senilis, unspecified eye | No | Anterior pole / Ocular surface |
| H18.429 | Band keratopathy, unspecified eye | No | Anterior pole / Ocular surface |
| H18.449 | Keratomalacia, unspecified eye | No | Anterior pole / Ocular surface |
| H18.459 | Nodular corneal degeneration, unspecified eye | No | Anterior pole / Ocular surface |
| H18.469 | Peripheral corneal degeneration, unspecified eye | No | Anterior pole / Ocular surface |
| H18.49 | Other corneal degeneration | No | Anterior pole / Ocular surface |
| H18.50 | Unspecified hereditary corneal dystrophies | No | Anterior pole / Ocular surface |
| H18.51 | Endothelial corneal dystrophy | No | Anterior pole / Ocular surface |
| H18.53 | Granular corneal dystrophy | No | Anterior pole / Ocular surface |
| H18.54 | Lattice corneal dystrophy | No | Anterior pole / Ocular surface |
| H18.55 | Macular corneal dystrophy | No | Anterior pole / Ocular surface |
| H18.59 | Other hereditary corneal dystrophies | No | Anterior pole / Ocular surface |
| H18.59 | Other hereditary corneal dystrophies | No | Anterior pole / Ocular surface |
| H18.609 | Keratoconus, unspecified, unspecified eye | No | Anterior pole / Ocular surface |
| H18.619 | Keratoconus, stable, unspecified eye | No | Anterior pole / Ocular surface |
| H18.629 | Keratoconus, unstable, unspecified eye | Yes | Anterior pole / Ocular surface |
| H18.719 | Corneal ectasia, unspecified eye | No | Anterior pole / Ocular surface |
| H18.729 | Corneal staphyloma, unspecified eye | No | Anterior pole / Ocular surface |
| H18.739 | Descemetocele, unspecified eye | Yes | Anterior pole / Ocular surface |
| **ICD‐10 Diagnosis Code** | **ICD-10 Diagnosis** | **Likely  Emergent?** | **Diagnosis group** |
| H18.799 | Other corneal deformities, unspecified eye | No | Anterior pole / Ocular surface |
| H18.819 | Anesthesia and hypoesthesia of cornea, unspecified eye | No | Anterior pole / Ocular surface |
| H18.829 | Corneal disorder due to contact lens, unspecified eye | No | Anterior pole / Ocular surface |
| H18.839 | Recurrent erosion of cornea, unspecified eye | Yes | Anterior pole / Ocular surface |
| H18.899 | Other specified disorders of cornea, unspecified eye | Could not determine | Anterior pole / Ocular surface |
| H18.9 | Unspecified disorder of cornea | Could not  determine | Anterior pole / Ocular surface |
| H20.00 | Unspecified acute and subacute iridocyclitis | Yes | Ocular Inflammation |
| H20.019 | Primary iridocyclitis, unspecified eye | Yes | Ocular Inflammation |
| H20.029 | Recurrent acute iridocyclitis, unspecified eye | No | Ocular Inflammation |
| H20.039 | Secondary infectious iridocyclitis, unspecified eye | Yes | Ocular Inflammation |
| H20.049 | Secondary noninfectious iridocyclitis, unspecified eye | Yes | Ocular Inflammation |
| H20.059 | Hypopyon, unspecified eye | Yes | Ocular Inflammation |
| H20.10 | Chronic iridocyclitis, unspecified eye | No | Ocular Inflammation |
| H20.20 | Lens-induced iridocyclitis, unspecified eye | No | Ocular Inflammation |
| H20.819 | Fuchs' heterochromic cyclitis, unspecified eye | No | Ocular Inflammation |
| H20.829 | Vogt-Koyanagi syndrome, unspecified eye | No | Ocular Inflammation |
| H20.9 | Unspecified iridocyclitis | Could not determine | Ocular Inflammation |
| H20.9 | Unspecified iridocyclitis | No | Ocular Inflammation |
| H21.00 | Hyphema, unspecified eye | Yes | Ocular Inflammation |
| H21.1X9 | Other vascular disorders of iris and ciliary body, unspecified eye | Yes | Ocular Inflammation |
| H21.239 | Degeneration of iris (pigmentary), unspecified eye | No | Ocular Inflammation |
| H21.249 | Degeneration of pupillary margin, unspecified eye | No | Ocular Inflammation |
| H21.259 | Iridoschisis, unspecified eye | No | Ocular Inflammation |
| H21.269 | Iris atrophy (essential) (progressive), unspecified eye | No | Ocular Inflammation |
| H21.279 | Miotic pupillary cyst, unspecified eye | No | Ocular Inflammation |
| H21.29 | Other iris atrophy | No | Ocular Inflammation |
| H21.309 | Idiopathic cysts of iris, ciliary body or anterior chamber, unspecified eye | No | Ocular Inflammation |
| H21.329 | Implantation cysts of iris, ciliary body or anterior chamber, unspecified eye | No | Ocular Inflammation |
| H21.349 | Primary cyst of pars plana, unspecified eye | No | Ocular Inflammation |
| H21.40 | Pupillary membranes, unspecified eye | No | Ocular Inflammation |
| **ICD‐10 Diagnosis Code** | **ICD-10 Diagnosis** | **Likely  Emergent?** | **Diagnosis group** |
| H21.509 | Unspecified adhesions of iris and ciliary body, unspecified eye | No | Ocular Inflammation |
| H21.519 | Anterior synechiae (iris), unspecified eye | No | Ocular Inflammation |
| H21.529 | Goniosynechiae, unspecified eye | No | Ocular Inflammation |
| H21.539 | Iridodialysis, unspecified eye | No | Ocular Inflammation |
| H21.549 | Posterior synechiae (iris), unspecified eye | No | Ocular Inflammation |
| H21.559 | Recession of chamber angle, unspecified eye | No | Ocular Inflammation |
| H21.569 | Pupillary abnormality, unspecified eye | No | Ocular Inflammation |
| H21.81 | Floppy iris syndrome | No | Ocular Inflammation |
| H21.82 | Plateau iris syndrome (post-iridectomy) (postprocedural) | No | Ocular Inflammation |
| H21.89 | Other specified disorders of iris and ciliary body | No | Ocular Inflammation |
| H21.9 | Unspecified disorder of iris and ciliary body | Could not determine | Ocular Inflammation |
| H25.019 | Cortical age-related cataract, unspecified eye | No | Anterior pole / Ocular surface |
| H25.039 | Anterior subcapsular polar age-related cataract, unspecified eye | No | Anterior pole / Ocular surface |
| H25.049 | Posterior subcapsular polar age-related cataract, unspecified eye | No | Anterior pole / Ocular surface |
| H25.099 | Other age-related incipient cataract, unspecified eye | No | Anterior pole / Ocular surface |
| H25.10 | Age-related nuclear cataract, unspecified eye | No | Anterior pole / Ocular surface |
| H25.20 | Age-related cataract, morgagnian type, unspecified eye | No | Anterior pole / Ocular surface |
| H25.819 | Combined forms of age-related cataract, unspecified eye | No | Anterior pole / Ocular surface |
| H25.89 | Other age-related cataract | No | Anterior pole / Ocular surface |
| H25.89 | Other age-related cataract | No | Anterior pole / Ocular surface |
| H25.9 | Unspecified age-related cataract | No | Anterior pole / Ocular surface |
| H26.009 | Unspecified infantile and juvenile cataract, unspecified eye | No | Anterior pole / Ocular surface |
| H26.039 | Infantile and juvenile nuclear cataract, unspecified eye | No | Anterior pole / Ocular surface |
| H26.049 | Anterior subcapsular polar infantile and juvenile cataract, unspecified eye | No | Anterior pole / Ocular surface |
| H26.059 | Posterior subcapsular polar infantile and juvenile cataract, unspecified eye | No | Anterior pole / Ocular surface |
| H26.109 | Unspecified traumatic cataract, unspecified eye | Yes | Anterior pole / Ocular surface |
| H26.119 | Localized traumatic opacities, unspecified eye | No | Anterior pole / Ocular surface |
| H26.129 | Partially resolved traumatic cataract, unspecified eye | No | Anterior pole / Ocular surface |
| H26.139 | Total traumatic cataract, unspecified eye | Yes | Anterior pole / Ocular surface |
| **ICD‐10 Diagnosis Code** | **ICD-10 Diagnosis** | **Likely  Emergent?** | **Diagnosis group** |
| H26.20 | Unspecified complicated cataract | No | Anterior pole / Ocular surface |
| H26.239 | Glaucomatous flecks (subcapsular), unspecified eye | No | Anterior pole / Ocular surface |
| H26.30 | Drug-induced cataract, unspecified eye | No | Anterior pole / Ocular surface |
| H26.40 | Unspecified secondary cataract | No | Anterior pole / Ocular surface |
| H26.499 | Other secondary cataract, unspecified eye | No | Anterior pole / Ocular surface |
| H26.499 | Other secondary cataract, unspecified eye | No | Anterior pole / Ocular surface |
| H26.8 | Other specified cataract | No | Anterior pole / Ocular surface |
| H26.8 | Other specified cataract | No | Anterior pole / Ocular surface |
| H26.9 | Unspecified cataract | No | Anterior pole / Ocular surface |
| H27.00 | Aphakia, unspecified eye | No | Anterior pole / Ocular surface |
| H27.119 | Subluxation of lens, unspecified eye | No | Anterior pole / Ocular surface |
| H27.129 | Anterior dislocation of lens, unspecified eye | Yes | Anterior pole / Ocular surface |
| H27.139 | Posterior dislocation of lens, unspecified eye | Yes | Anterior pole / Ocular surface |
| H27.8 | Other specified disorders of lens | Could not | Anterior pole / Ocular surface |
| H30.009 | Unspecified focal chorioretinal inflammation, unspecified eye | No | Ocular Inflammation |
| H30.019 | Focal chorioretinal inflammation, juxtapapillary, unspecified eye | Yes | Ocular Inflammation |
| H30.029 | Focal chorioretinal inflammation of posterior pole, unspecified eye | Could not determine | Ocular Inflammation |
| H30.029 | Focal chorioretinal inflammation of posterior pole, unspecified eye | Yes | Ocular Inflammation |
| H30.109 | Unspecified disseminated chorioretinal inflammation, unspecified eye | Could not determine | Ocular Inflammation |
| H30.119 | Disseminated chorioretinal inflammation of posterior pole, unspecified eye | Could not determine | Ocular Inflammation |
| H30.139 | Disseminated chorioretinal inflammation, generalized, unspecified eye | Could not  determine | Ocular Inflammation |
| H30.149 | Acute posterior multifocal placoid pigment epitheliopathy, unspecified eye | Yes | Ocular Inflammation |
| H30.819 | Harada's disease, unspecified eye | No | Ocular Inflammation |
| H30.90 | Unspecified chorioretinal inflammation, unspecified eye | Could not  determine | Ocular Inflammation |
| H31.009 | Unspecified chorioretinal scars, unspecified eye | No | Retina |
| H31.019 | Macula scars of posterior pole (postinflammatory) (post-traumatic), unspecified eye | No | Retina |
| H31.029 | Solar retinopathy, unspecified eye | No | Retina |
| H31.109 | Choroidal degeneration, unspecified, unspecified eye | No | Retina |
| **ICD‐10 Diagnosis Code** | **ICD-10 Diagnosis** | **Likely  Emergent?** | **Diagnosis group** |
| H31.119, H31.129, H31.109 | Age-related choroidal atrophy, unspecified eye, Diffuse secondary atrophy of choroid, unspecified eye, Choroidal degeneration, unspecified, unspecified eye | No | Retina |
| H31.129 | Diffuse secondary atrophy of choroid, unspecified eye | No | Retina |
| H31.309 | Unspecified choroidal hemorrhage, unspecified eye | Yes | Retina |
| H31.319 | Expulsive choroidal hemorrhage, unspecified eye | Yes | Retina |
| H31.329 | Choroidal rupture, unspecified eye | Yes | Retina |
| H31.409 | Unspecified choroidal detachment, unspecified eye | Yes | Retina |
| H31.419 | Hemorrhagic choroidal detachment, unspecified eye | Yes | Retina |
| H31.429 | Serous choroidal detachment, unspecified eye | No | Retina |
| H31.8 | Other specified disorders of choroid | No | Retina |
| H31.9 | Unspecified disorder of choroid | No | Retina |
| H33.00 | Unspecified retinal detachment with retinal break | Yes | Retina |
| H33.009 | Unspecified retinal detachment with retinal break, unspecified eye | Yes | Retina |
| H33.019 | Retinal detachment with single break, unspecified eye | Yes | Retina |
| H33.029 | Retinal detachment with multiple breaks, unspecified eye | Yes | Retina |
| H33.039 | Retinal detachment with giant retinal tear, unspecified eye | Yes | Retina |
| H33.049 | Retinal detachment with retinal dialysis, unspecified eye | Yes | Retina |
| H33.059 | Total retinal detachment, unspecified eye | Yes | Retina |
| H33.059 | Total retinal detachment, unspecified eye | No | Retina |
| H33.109 | Unspecified retinoschisis, unspecified eye | No | Retina |
| H33.199 | Other retinoschisis and retinal cysts, unspecified eye | No | Retina |
| H33.199 | Other retinoschisis and retinal cysts, unspecified eye | No | Retina |
| H33.199 | Other retinoschisis and retinal cysts, unspecified eye | No | Retina |
| H33.309 | Unspecified retinal break, unspecified eye | Yes | Retina |
| H33.319 | Horseshoe tear of retina without detachment, unspecified eye | Yes | Retina |
| H33.329 | Round hole, unspecified eye | No | Retina |
| H33.339 | Multiple defects of retina without detachment, unspecified eye | Yes | Retina |
| H33.40 | Traction detachment of retina, unspecified eye | No | Retina |
| H33.8 | Other retinal detachments | Could not determine | Retina |
| **ICD‐10 Diagnosis Code** | **ICD-10 Diagnosis** | **Likely  Emergent?** | **Diagnosis group** |
| H33.8 | Other retinal detachments | No | Retina |
| H34.00 | Transient retinal artery occlusion, unspecified eye | Yes | Retina |
| H34.10 | Central retinal artery occlusion, unspecified eye | Yes | Retina |
| H34.219 | Partial retinal artery occlusion, unspecified eye | Yes | Retina |
| H34.239 | Retinal artery branch occlusion, unspecified eye | Yes | Retina |
| H34.81 | Central retinal vein occlusion | No | Retina |
| H34.829 | Venous engorgement, unspecified eye | No | Retina |
| H34.83 | Tributary (branch) retinal vein occlusion | No | Retina |
| H34.9 | Unspecified retinal vascular occlusion | Could not determine | Retina |
| H35.00 | Unspecified background retinopathy | No | Retina |
| H35.019 | Changes in retinal vascular appearance, unspecified eye | No | Retina |
| H35.029 | Exudative retinopathy, unspecified eye | No | Retina |
| H35.039 | Hypertensive retinopathy, unspecified eye | No | Retina |
| H35.049 | Retinal micro-aneurysms, unspecified, unspecified eye | No | Retina |
| H35.059 | Retinal neovascularization, unspecified, unspecified eye | No | Retina |
| H35.069 | Retinal vasculitis, unspecified eye | No | Retina |
| H35.079 | Retinal telangiectasis, unspecified eye | No | Retina |
| H35.09 | Other intraretinal microvascular abnormalities | No | Retina |
| H35.109 | Retinopathy of prematurity, unspecified, unspecified eye | No | Retina |
| H35.119 | Retinopathy of prematurity, stage 0, unspecified eye | No | Retina |
| H35.139 | Retinopathy of prematurity, stage 2, unspecified eye | No | Retina |
| H35.149 | Retinopathy of prematurity, stage 3, unspecified eye | No | Retina |
| H35.159 | Retinopathy of prematurity, stage 4, unspecified eye | No | Retina |
| H35.169 | Retinopathy of prematurity, stage 5, unspecified eye | No | Retina |
| H35.179 | Retrolental fibroplasia, unspecified eye | No | Retina |
| H35.20 | Other non-diabetic proliferative retinopathy, unspecified eye | No | Retina |
| H35.30 | Unspecified macular degeneration | No | Retina |
| H35.3190 | Nonexudative age-related macular degeneration, unspecified eye, stage unspecified | No | Retina |
| H35.32 | Exudative age-related macular degeneration | No | Retina |
| H35.33 | Angioid streaks of macula | No | Retina |
| **ICD‐10 Diagnosis Code** | **ICD-10 Diagnosis** | **Likely  Emergent?** | **Diagnosis group** |
| H35.349 | Macular cyst, hole, or pseudohole, unspecified eye | No | Retina |
| H35.359 | Cystoid macular degeneration, unspecified eye | No | Retina |
| H35.379 | Puckering of macula, unspecified eye | No | Retina |
| H35.389 | Toxic maculopathy, unspecified eye | No | Retina |
| H35.40 | Unspecified peripheral retinal degeneration | No | Retina |
| H35.419 | Lattice degeneration of retina, unspecified eye | No | Retina |
| H35.449 | Age-related reticular degeneration of retina, unspecified eye | No | Retina |
| H35.50 | Unspecified hereditary retinal dystrophy | No | Retina |
| H35.51 | Vitreoretinal dystrophy | No | Retina |
| H35.52 | Pigmentary retinal dystrophy | No | Retina |
| H35.53 | Other dystrophies primarily involving the sensory retina | No | Retina |
| H35.54 | Dystrophies primarily involving the retinal pigment epithelium | No | Retina |
| H35.60 | Retinal hemorrhage, unspecified eye | Could not determine | Retina |
| H35.719 | Central serous chorioretinopathy, unspecified eye | No | Retina |
| H35.729 | Serous detachment of retinal pigment epithelium, unspecified eye | No | Retina |
| H35.739 | Hemorrhagic detachment of retinal pigment epithelium, unspecified eye | Yes | Retina |
| H35.81 | Retinal edema | No | Retina |
| H35.82 | Retinal ischemia | Yes | Retina |
| H35.89 | Other specified retinal disorders | No | Retina |
| H35.89 | Other specified retinal disorders | No | Retina |
| H35.89 | Other specified retinal disorders | No | Retina |
| H35.9 | Unspecified retinal disorder | Could not  determine | Retina |
| H40 | Glaucoma | Could not  determine | Glaucoma |
| H40.009 | Preglaucoma, unspecified, unspecified eye | No | Glaucoma |
| H40.019 | Open angle with borderline findings, low risk, unspecified eye | No | Glaucoma |
| H40.039 | Anatomical narrow angle, unspecified eye | No | Glaucoma |
| H40.049 | Steroid responder, unspecified eye | No | Glaucoma |
| H40.059 | Ocular hypertension, unspecified eye | No | Glaucoma |
| H40.10 | Unspecified open-angle glaucoma | No | Glaucoma |
| H40.1194 | Primary open-angle glaucoma, unspecified eye, indeterminate stage | No | Glaucoma |
| **ICD‐10 Diagnosis Code** | **ICD-10 Diagnosis** | **Likely  Emergent?** | **Diagnosis group** |
| H40.1290 | Low-tension glaucoma, unspecified eye, stage unspecified | No | Glaucoma |
| H40.1390 | Pigmentary glaucoma, unspecified eye, stage unspecified | Yes | Glaucoma |
| H40.1490 | Capsular glaucoma with pseudoexfoliation of lens, unspecified eye, stage unspecified | No | Glaucoma |
| H40.159 | Residual stage of open-angle glaucoma, unspecified eye | No | Glaucoma |
| H40.20 | Unspecified primary angle-closure glaucoma | No | Glaucoma |
| H40.219 | Acute angle-closure glaucoma, unspecified eye | Yes | Glaucoma |
| H40.2290 | Chronic angle-closure glaucoma, unspecified eye, stage unspecified | No | Glaucoma |
| H40.239 | Intermittent angle-closure glaucoma, unspecified eye | Yes | Glaucoma |
| H40.249 | Residual stage of angle-closure glaucoma, unspecified eye | No | Glaucoma |
| H40.30X4 | Glaucoma secondary to eye trauma, unspecified eye, indeterminate stage | Yes | Glaucoma |
| H40.40 | Glaucoma secondary to eye inflammation, unspecified eye | Yes | Glaucoma |
| H40.40X4 | Glaucoma secondary to eye inflammation, unspecified eye, indeterminate stage | Yes | Glaucoma |
| H40.50 | Glaucoma secondary to other eye disorders, unspecified eye | No | Glaucoma |
| H40.50 | Glaucoma secondary to other eye disorders, unspecified eye | Yes | Glaucoma |
| H40.50 | Glaucoma secondary to other eye disorders, unspecified eye | Could not determine | Glaucoma |
| H40.50 | Glaucoma secondary to other eye disorders, unspecified eye | Could not determine | Glaucoma |
| H40.60X0 | Glaucoma secondary to drugs, unspecified eye, stage unspecified | No | Glaucoma |
| H40.819 | Glaucoma with increased episcleral venous pressure, unspecified eye | Could not determine | Glaucoma |
| H40.829 | Hypersecretion glaucoma, unspecified eye | No | Glaucoma |
| H40.89 | Other specified glaucoma | Could not determine | Glaucoma |
| H40.89 | Other specified glaucoma | Could not  determine | Glaucoma |
| H40.89 | Other specified glaucoma | Yes | Glaucoma |
| H40.89 | Other specified glaucoma | No | Glaucoma |
| H40.89 | Other specified glaucoma | No | Glaucoma |
| H40.9 | Unspecified glaucoma | Could not determine | Glaucoma |
| H42 | Glaucoma in diseases classified elsewhere | No | Glaucoma |
| H43.00 | Vitreous prolapse, unspecified eye | Yes | Retina |
| H43.10 | Vitreous hemorrhage, unspecified eye | Yes | Retina |
| H43.20 | Crystalline deposits in vitreous body, unspecified eye | No | Retina |
| **ICD‐10 Diagnosis Code** | **ICD-10 Diagnosis** | **Likely  Emergent?** | **Diagnosis group** |
| H43.319 | Vitreous membranes and strands, unspecified eye | No | Retina |
| H43.399 | Other vitreous opacities, unspecified eye | No | Retina |
| H43.819 | Vitreous degeneration, unspecified eye | No | Retina |
| H43.819 | Vitreous degeneration, unspecified eye | Could not determine | Retina |
| H43.9 | Unspecified disorder of vitreous body | Could not  determine | Retina |
| H44.009 | Unspecified purulent endophthalmitis, unspecified eye | Yes | Retina |
| H44.009 | Unspecified purulent endophthalmitis, unspecified eye | Yes | Retina |
| H44.009 | Unspecified purulent endophthalmitis, unspecified eye | No | Retina |
| H44.019 | Panophthalmitis (acute), unspecified eye | Yes | Retina |
| H44.119 | Panuveitis, unspecified eye | Yes | Retina |
| H44.139 | Sympathetic uveitis, unspecified eye | Yes | Retina |
| H44.19 | Other endophthalmitis | Yes | Retina |
| H44.20 | Degenerative myopia, unspecified eye | No | Miscellany |
| H44.319 | Chalcosis, unspecified eye | Could not determine | Miscellany |
| H44.329 | Siderosis of eye, unspecified eye | Could not determine | Miscellany |
| H44.399 | Other degenerative disorders of globe, unspecified eye | Could not determine | Miscellany |
| H44.40 | Unspecified hypotony of eye | Could not  determine | Miscellany |
| H44.419 | Flat anterior chamber hypotony of unspecified eye | Yes | Miscellany |
| H44.429 | Hypotony of unspecified eye due to ocular fistula | Yes | Miscellany |
| H44.439 | Hypotony of eye due to other ocular disorders, unspecified eye | Could not determine | Miscellany |
| H44.449 | Primary hypotony of unspecified eye | Could not determine | Miscellany |
| H44.50 | Unspecified degenerated conditions of globe | Could not determine | Miscellany |
| H44.519 | Absolute glaucoma, unspecified eye | No | Glaucoma |
| H44.529 | Atrophy of globe, unspecified eye | No | Miscellany |
| H44.539 | Leucocoria, unspecified eye | No | Miscellany |
| H44.619 | Retained (old) magnetic foreign body in anterior chamber, unspecified eye | Yes | Miscellany |
| H44.629 | Retained (old) magnetic foreign body in iris or ciliary body, unspecified eye | Yes | Miscellany |
| H44.639 | Retained (old) magnetic foreign body in lens, unspecified eye | Yes | Miscellany |
| H44.649 | Retained (old) magnetic foreign body in posterior wall of globe, unspecified eye | Yes | Miscellany |
| **ICD‐10 Diagnosis Code** | **ICD-10 Diagnosis** | **Likely  Emergent?** | **Diagnosis group** |
| H44.699 | Retained (old) intraocular foreign body, magnetic, in other or multiple sites, unspecified eye | Yes | Miscellany |
| H44.709 | Unspecified retained (old) intraocular foreign body, nonmagnetic, unspecified eye | Yes | Miscellany |
| H44.719 | Retained (nonmagnetic) (old) foreign body in anterior chamber, unspecified eye | Yes | Miscellany |
| H44.739 | Retained (nonmagnetic) (old) foreign body in lens, unspecified eye | Yes | Miscellany |
| H44.749 | Retained (nonmagnetic) (old) foreign body in posterior wall of globe, unspecified eye | Yes | Miscellany |
| H44.759 | Retained (nonmagnetic) (old) foreign body in vitreous body, unspecified eye | Yes | Miscellany |
| H44.799 | Retained (old) intraocular foreign body, nonmagnetic, in other or multiple sites, unspecified eye | Yes | Miscellany |
| H44.819 | Hemophthalmos, unspecified eye | Could not determine | Miscellany |
| H44.829 | Luxation of globe, unspecified eye | Yes | Oculoplastics / Orbit |
| H44.89 | Other disorders of globe | Could not determine | Miscellany |
| H46.00 | Optic papillitis, unspecified eye | Yes | Neuroophthalmology |
| H46.10 | Retrobulbar neuritis, unspecified eye | Yes | Neuroophthalmology |
| H46.2 | Nutritional optic neuropathy | No | Neuroophthalmology |
| H46.3 | Toxic optic neuropathy | No | Neuroophthalmology |
| H46.8 | Other optic neuritis | Could not determine | Neuroophthalmology |
| H46.9 | Unspecified optic neuritis | Could not determine | Neuroophthalmology |
| H47.019 | Ischemic optic neuropathy, unspecified eye | No | Neuroophthalmology |
| H47.029 | Hemorrhage in optic nerve sheath, unspecified eye | Yes | Neuroophthalmology |
| H47.039 | Optic nerve hypoplasia, unspecified eye | No | Neuroophthalmology |
| H47.099 | Other disorders of optic nerve, not elsewhere classified, unspecified eye | Could not determine | Neuroophthalmology |
| H47.10 | Unspecified papilledema | Could not determine | Neuroophthalmology |
| H47.11 | Papilledema associated with increased intracranial pressure | Yes | Neuroophthalmology |
| H47.12 | Papilledema associated with decreased ocular pressure | No | Neuroophthalmology |
| H47.13 | Papilledema associated with retinal disorder | No | Neuroophthalmology |
| H47.149 | Foster-Kennedy syndrome, unspecified eye | No | Neuroophthalmology |
| H47.20 | Unspecified optic atrophy | No | Neuroophthalmology |
| H47.219 | Primary optic atrophy, unspecified eye | No | Neuroophthalmology |
| H47.22 | Hereditary optic atrophy | No | Neuroophthalmology |
| H47.239 | Glaucomatous optic atrophy, unspecified eye | No | Neuroophthalmology |
| **ICD‐10 Diagnosis Code** | **ICD-10 Diagnosis** | **Likely  Emergent?** | **Diagnosis group** |
| H47.299 | Other optic atrophy, unspecified eye | No | Neuroophthalmology |
| H47.299 | Other optic atrophy, unspecified eye | No | Neuroophthalmology |
| H47.319 | Coloboma of optic disc, unspecified eye | No | Neuroophthalmology |
| H47.329 | Drusen of optic disc, unspecified eye | No | Neuroophthalmology |
| H47.339 | Pseudopapilledema of optic disc, unspecified eye | No | Neuroophthalmology |
| H47.399 | Other disorders of optic disc, unspecified eye | No | Neuroophthalmology |
| H47.41 | Disorders of optic chiasm in (due to) inflammatory disorders | Could not  determine | Neuroophthalmology |
| H47.42 | Disorders of optic chiasm in (due to) neoplasm | Could not  determine | Neuroophthalmology |
| H47.43 | Disorders of optic chiasm in (due to) vascular disorders | Could not determine | Neuroophthalmology |
| H47.49 | Disorders of optic chiasm in (due to) other disorders | Could not determine | Neuroophthalmology |
| H47.519 | Disorders of visual pathways in (due to) inflammatory disorders, unspecified side | Could not determine | Neuroophthalmology |
| H47.539 | Disorders of visual pathways in (due to) vascular disorders, unspecified side | Could not determine | Neuroophthalmology |
| H47.619 | Cortical blindness, unspecified side of brain | Could not determine | Neuroophthalmology |
| H47.629 | Disorders of visual cortex in (due to) inflammatory disorders, unspecified side of brain | Could not determine | Neuroophthalmology |
| H47.649 | Disorders of visual cortex in (due to) vascular disorders, unspecified side of brain | Could not determine | Neuroophthalmology |
| H47.9 | Unspecified disorder of visual pathways | Could not determine | Neuroophthalmology |
| H49.00 | Third [oculomotor] nerve palsy, unspecified eye | Yes | Neuroophthalmology |
| H49.00 | Third [oculomotor] nerve palsy, unspecified eye | Yes | Neuroophthalmology |
| H49.10 | Fourth [trochlear] nerve palsy, unspecified eye | Yes | Neuroophthalmology |
| H49.20 | Sixth [abducent] nerve palsy, unspecified eye | Yes | Neuroophthalmology |
| H49.30 | Total (external) ophthalmoplegia, unspecified eye | Yes | Neuroophthalmology |
| H49.40 | Progressive external ophthalmoplegia, unspecified eye | Yes | Neuroophthalmology |
| H49.889 | Other paralytic strabismus, unspecified eye | No | Neuroophthalmology |
| H50.00 | Unspecified esotropia | Could not determine | Neuroophthalmology |
| H50.01 | Monocular esotropia | Could not  determine | Neuroophthalmology |
| H50.02 | Monocular esotropia with A pattern | No | Neuroophthalmology |
| H50.04 | Monocular esotropia with other noncomitancies | No | Neuroophthalmology |
| H50.05 | Alternating esotropia | No | Neuroophthalmology |
| H50.06 | Alternating esotropia with A pattern | No | Neuroophthalmology |
| **ICD‐10 Diagnosis Code** | **ICD-10 Diagnosis** | **Likely  Emergent?** | **Diagnosis group** |
| H50.08 | Alternating esotropia with other noncomitancies | No | Neuroophthalmology |
| H50.10 | Unspecified exotropia | Could not determine | Neuroophthalmology |
| H50.11 | Monocular exotropia | Could not determine | Neuroophthalmology |
| H50.12 | Monocular exotropia with A pattern | No | Neuroophthalmology |
| H50.13 | Monocular exotropia with V pattern | No | Neuroophthalmology |
| H50.15 | Alternating exotropia | No | Neuroophthalmology |
| H50.17 | Alternating exotropia with V pattern | No | Neuroophthalmology |
| H50.2 | Vertical strabismus | No | Neuroophthalmology |
| H50.2 | Vertical strabismus | Could not determine | Neuroophthalmology |
| H50.30 | Unspecified intermittent heterotropia | No | Neuroophthalmology |
| H50.31 | Intermittent monocular esotropia | No | Neuroophthalmology |
| H50.32 | Intermittent alternating esotropia | No | Neuroophthalmology |
| H50.33 | Intermittent monocular exotropia | Could not determine | Neuroophthalmology |
| H50.34 | Intermittent alternating exotropia | No | Neuroophthalmology |
| H50.40 | Unspecified heterotropia | No | Neuroophthalmology |
| H50.43 | Accommodative component in esotropia | No | Neuroophthalmology |
| H50.50 | Unspecified heterophoria | No | Neuroophthalmology |
| H50.51 | Esophoria | No | Neuroophthalmology |
| H50.52 | Exophoria | No | Neuroophthalmology |
| H50.53 | Vertical heterophoria | Could not  determine | Neuroophthalmology |
| H50.60 | Mechanical strabismus, unspecified | No | Neuroophthalmology |
| H50.61 | Brown's sheath syndrome | No | Neuroophthalmology |
| H50.69 | Other mechanical strabismus | No | Neuroophthalmology |
| H50.69 | Other mechanical strabismus | No | Neuroophthalmology |
| H50.81 | Duane's syndrome | No | Neuroophthalmology |
| H50.89 | Other specified strabismus | Could not  determine | Neuroophthalmology |
| H50.89 | Other specified strabismus | Yes | Neuroophthalmology |
| H51.0 | Palsy (spasm) of conjugate gaze | Yes | Neuroophthalmology |
| H51.0 | Palsy (spasm) of conjugate gaze | No | Neuroophthalmology |
| H51.11 | Convergence insufficiency | Could not  determine | Neuroophthalmology |
| H51.12 | Convergence excess | Could not  determine | Neuroophthalmology |
| **ICD‐10 Diagnosis Code** | **ICD-10 Diagnosis** | **Likely  Emergent?** | **Diagnosis group** |
| H51.20 | Internuclear ophthalmoplegia, unspecified eye | Yes | Neuroophthalmology |
| H51.8 | Other specified disorders of binocular movement | Yes | Neuroophthalmology |
| H51.8 | Other specified disorders of binocular movement | Could not determine | Neuroophthalmology |
| H51.9, H50.9 | Unspecified disorder of binocular movement | Could not determine | Neuroophthalmology |
| H52.00 | Hypermetropia, unspecified eye | No | Miscellany |
| H52.10 | Myopia, unspecified eye | No | Miscellany |
| H52.209 | Unspecified astigmatism, unspecified eye | No | Miscellany |
| H52.219 | Irregular astigmatism, unspecified eye | No | Miscellany |
| H52.229 | Regular astigmatism, unspecified eye | No | Miscellany |
| H52.31 | Anisometropia | No | Miscellany |
| H52.32 | Aniseikonia | Yes | Miscellany |
| H52.4 | Presbyopia | No | Miscellany |
| H52.519 | Internal ophthalmoplegia (complete) (total), unspecified eye | No | Neuroophthalmology |
| H52.529 | Paresis of accommodation, unspecified eye | Could not | Anterior pole / Ocular surface |
| H52.539 | Spasm of accommodation, unspecified eye | No | Anterior pole / Ocular surface |
| H52.6 | Other disorders of refraction | No | Anterior pole / Ocular surface |
| H52.6 | Other disorders of refraction | No | Anterior pole / Ocular surface |
| H52.7 | Unspecified disorder of refraction | No | Anterior pole / Ocular surface |
| H53.009 | Unspecified amblyopia, unspecified eye | No | Miscellany |
| H53.019 | Deprivation amblyopia, unspecified eye | No | Miscellany |
| H53.029 | Refractive amblyopia, unspecified eye | No | Anterior pole / Ocular surface |
| H53.039 | Strabismic amblyopia, unspecified eye | No | Neuroophthalmology |
| H53.10 | Unspecified subjective visual disturbances | No | Miscellany |
| H53.129 | Transient visual loss, unspecified eye | Yes | Neuroophthalmology |
| H53.139 | Sudden visual loss, unspecified eye | Yes | Neuroophthalmology |
| H53.149 | Visual discomfort, unspecified | No | Miscellany |
| H53.15 | Visual distortions of shape and size | No | Miscellany |
| H53.16, R48.3, R44.1 | Psychophysical visual disturbances, Visual agnosia, Visual hallucinations | No | Neuroophthalmology |
| H53.19 | Other subjective visual disturbances | No | Miscellany |
| H53.2 | Diplopia | Could not determine | Neuroophthalmology |
| **ICD‐10 Diagnosis Code** | **ICD-10 Diagnosis** | **Likely  Emergent?** | **Diagnosis group** |
| H53.30 | Unspecified disorder of binocular vision | Could not determine | Neuroophthalmology |
| H53.31 | Abnormal retinal correspondence | No | Retina |
| H53.33 | Simultaneous visual perception without fusion | Could not  determine | Neuroophthalmology |
| H53.34 | Suppression of binocular vision | Could not  determine | Neuroophthalmology |
| H53.40 | Unspecified visual field defects | Could not determine | Neuroophthalmology |
| H53.419 | Scotoma involving central area, unspecified eye | Yes | Neuroophthalmology |
| H53.429 | Scotoma of blind spot area, unspecified eye | Yes | Neuroophthalmology |
| H53.439 | Sector or arcuate defects, unspecified eye | Could not  determine | Neuroophthalmology |
| H53.459 | Other localized visual field defect, unspecified eye | Could not determine | Neuroophthalmology |
| H53.469 | Homonymous bilateral field defects, unspecified side | Yes | Neuroophthalmology |
| H53.47 | Heteronymous bilateral field defects | Could not determine | Neuroophthalmology |
| H53.489 | Generalized contraction of visual field, unspecified eye | Could not  determine | Neuroophthalmology |
| H53.51 | Achromatopsia | No | Miscellany |
| H53.52 | Acquired color vision deficiency | No | Miscellany |
| H53.59 | Other color vision deficiencies | No | Miscellany |
| H53.60 | Unspecified night blindness | No | Miscellany |
| H53.62 | Acquired night blindness | No | Miscellany |
| H53.8 | Other visual disturbances | Could not  determine | Miscellany |
| H53.9 | Unspecified visual disturbance | Could not  determine | Miscellany |
| H53.9 | Unspecified visual disturbance | Could not determine | Miscellany |
| H54.0 | Blindness and low vision | Could not determine | Miscellany |
| H54.0 | Blindness, both eyes | Could not determine | Miscellany |
| H54.0 | Blindness, both eyes, different category levels | Could not determine | Miscellany |
| H54.0X | Blindness, both eyes, different category levels | Could not determine | Miscellany |
| H54.1 | Blindness, one eye, low vision other eye | Could not determine | Miscellany |
| H54.1 | Blindness, one eye, low vision other eye | Could not determine | Miscellany |
| H54.1 | Blindness, one eye, low vision other eye | Could not determine | Miscellany |
| H54.10 | Blindness, one eye, low vision other eye, unspecified eyes | Could not determine | Miscellany |
| H54.10 | Blindness, one eye, low vision other eye, unspecified eyes | Could not determine | Miscellany |
| **ICD‐10 Diagnosis Code** | **ICD-10 Diagnosis** | **Likely  Emergent?** | **Diagnosis group** |
| H54.2X | Low vision, both eyes, different category levels | Could not  determine | Miscellany |
| H54.2X | Low vision, both eyes, different category levels | Could not determine | Miscellany |
| H54.2X | Low vision, both eyes, different category levels | Could not determine | Miscellany |
| H54.2X | Low vision, both eyes, different category levels | Could not determine | Miscellany |
| H54.3 | Unqualified visual loss, both eyes | Could not determine | Miscellany |
| H54.40 | Blindness, one eye, unspecified eye | Could not determine | Miscellany |
| H54.40 | Blindness, one eye, unspecified eye | Could not determine | Miscellany |
| H54.40 | Blindness, one eye, unspecified eye | Could not  determine | Miscellany |
| H54.40 | Blindness, one eye, unspecified eye | Could not  determine | Miscellany |
| H54.40 | Blindness, one eye, unspecified eye | Could not  determine | Miscellany |
| H54.40 | Blindness, one eye, unspecified eye | Could not  determine | Miscellany |
| H54.40 | Blindness, one eye, unspecified eye | Could not determine | Miscellany |
| H54.40 | Blindness, one eye, unspecified eye | Could not determine | Miscellany |
| H54.40 | Blindness, one eye, unspecified eye | Could not determine | Miscellany |
| H54.50 | Low vision, one eye, unspecified eye | Could not determine | Miscellany |
| H54.50 | Low vision, one eye, unspecified eye | Could not determine | Miscellany |
| H54.50 | Low vision, one eye, unspecified eye | Could not determine | Miscellany |
| H54.50 | Low vision, one eye, unspecified eye | Could not determine | Miscellany |
| H54.50 | Low vision, one eye, unspecified eye | Could not determine | Miscellany |
| H54.50 | Low vision, one eye, unspecified eye | Could not determine | Miscellany |
| H54.50 | Low vision, one eye, unspecified eye | Could not determine | Miscellany |
| H54.50 | Low vision, one eye, unspecified eye | Could not determine | Miscellany |
| H54.50 | Low vision, one eye, unspecified eye | Could not determine | Miscellany |
| H54.60 | Unqualified visual loss, one eye, unspecified | Could not determine | Miscellany |
| H54.7 | Unspecified visual loss | Could not determine | Miscellany |
| H54.8 | Legal blindness, as defined in USA | Could not determine | Miscellany |
| H5441, H5442 | One eye: total impairment; other eye: normal vision | Could not determine | Miscellany |
| H55.00 | Unspecified nystagmus | Could not determine | Neuroophthalmology |
| **ICD‐10 Diagnosis Code** | **ICD-10 Diagnosis** | **Likely  Emergent?** | **Diagnosis group** |
| H55.01 | Congenital nystagmus | No | Neuroophthalmology |
| H55.02 | Latent nystagmus | No | Neuroophthalmology |
| H55.03 | Visual deprivation nystagmus | No | Neuroophthalmology |
| H55.09 | Other forms of nystagmus | Could not  determine | Neuroophthalmology |
| H55.09 | Other forms of nystagmus | No | Neuroophthalmology |
| H55.81 | Saccadic eye movements | Could not determine | Neuroophthalmology |
| H55.89 | Other irregular eye movements | Yes | Neuroophthalmology |
| H57.00 | Unspecified anomaly of pupillary function | Could not  determine | Neuroophthalmology |
| H57.02 | Anisocoria | Could not determine | Neuroophthalmology |
| H57.03 | Miosis | Could not determine | Neuroophthalmology |
| H57.04 | Mydriasis | Could not determine | Neuroophthalmology |
| H57.059 | Tonic pupil, unspecified eye | No | Neuroophthalmology |
| H57.09 | Other anomalies of pupillary function | No | Neuroophthalmology |
| H57.10 | Ocular pain, unspecified eye | Could not  determine | Miscellany |
| H57.89 | Other specified disorders of eye and adnexa | Could not determine | Miscellany |
| H57.9 | Unspecified disorder of eye and adnexa | Could not determine | Miscellany |
| H59.02 | Cataract (lens) fragments in eye following cataract surgery | Yes | Miscellany |
| H59.40 | Inflammation (infection) of postprocedural bleb, unspecified | Yes | Glaucoma |
| H59.41 | Inflammation (infection) of postprocedural bleb, stage 1 | Yes | Glaucoma |
| H59.42 | Inflammation (infection) of postprocedural bleb, stage 2 | Yes | Glaucoma |
| H59.43 | Inflammation (infection) of postprocedural bleb, stage 3 | Yes | Glaucoma |
| H21.89 | Other specified disorders of iris and ciliary body | No | Ocular Inflammation |
| Q15.0 | Congenital glaucoma | Yes | Glaucoma |
| T20.10 | Burn of first degree of head, face and neck, unespecified site | Yes | Trauma |
| T20.20 | Burn of second degree of head, face and neck, unespecified site | Yes | Trauma |
| S00.10 | Contusion of unspecified eyelid and periocular area | Yes | Trauma |
| S00.10 | Contusion of unspecified eyelid and periocular area | Yes | Trauma |
| S00.2 | Other and unspecified superficial injuries of eyelid and periocular area | Yes | Trauma |
| S01.109 | Unspecified open wound of unspecified eyelid and periocular area | Yes | Trauma |
| **ICD‐10 Diagnosis Code** | **ICD-10 Diagnosis** | **Likely  Emergent?** | **Diagnosis group** |
| S01.109A | Unspecified open wound of unspecified eyelid and periocular area | Yes | Trauma |
| S01.111 | Laceration without foreign body of right eyelid and periocular area, | Yes | Trauma |
| S01.119, S01.129 | Laceration with (S01.129) or without (S01.119) foreign body of unspecified eyelid and periocular area | Yes | Trauma |
| S01.119, S01.129 | Laceration with (S01.129) or without (S01.119) foreign body of unspecified eyelid and periocular area | Yes | Trauma |
| S02.3 | Fracture of orbital floor | Yes | Trauma |
| S02.30 | Fracture of orbital floor, unspecified side, open fracture | Yes | Trauma |
| S04.019 | Injury of optic nerve, unspecified eye | Yes | Trauma |
| S04.019 | Injury of optic nerve, unspecified eye | Yes | Trauma |
| S04.02 | Injury of optic chiasm | Yes | Trauma |
| S04.039 | Injury of optic tract and pathways, unspecified side | Yes | Trauma |
| S04.049 | Injury of visual cortex, unspecified side | Yes | Trauma |
| S04.10 | Injury of oculomotor nerve, unspecified side | Yes | Trauma |
| S05.0 | Injury of conjunctiva and corneal abrasion without foreign body, unspecified eye | Yes | Trauma |
| T86.840 | Corneal transplant rejection | Yes | Miscellany |
| S05.00 | Injury of conjunctiva and corneal abrasion without foreign body, unspecified eye | Yes | Trauma |
| S05.10 | Contusion of eyeball and orbital tissues, unspecified eye | Yes | Trauma |
| S05.10 | Contusion of eyeball and orbital tissues, unspecified eye | Yes | Trauma |
| S05.20 | Ocular laceration and rupture with prolapse or loss of intraocular tissue, unspecified eye | Yes | Trauma |
| S05.20 | Ocular laceration and rupture with prolapse or loss of intraocular tissue, unspecified eye | Yes | Trauma |
| S05.30 | Ocular laceration without prolapse or loss of intraocular tissue, unspecified eye | Yes | Trauma |
| S05.30 | Ocular laceration without prolapse or loss of intraocular tissue, unspecified eye | Yes | Trauma |
| S05.40 | Penetrating wound of orbit with or without foreign body | Yes | Trauma |
| S05.40 | Penetrating wound of orbit with or without foreign body, unspecified eye | Yes | Trauma |
| S05.50 | Penetrating wound with foreign body of unspecified eyeball | Yes | Trauma |
| S05.50 | Penetrating wound with foreign body of unspecified eyeball | Yes | Trauma |
| S05.60 | Penetrating wound without foreign body of unspecified eyeball | Yes | Trauma |
| S05.70 | Avulsion of unspecified eye | Yes | Trauma |
| S05.90 | Unspecified injury of unspecified eye and orbit, | Yes | Trauma |
| **ICD‐10 Diagnosis Code** | **ICD-10 Diagnosis** | **Likely  Emergent?** | **Diagnosis group** |
| S05.90 | Unspecified injury of unspecified eye and orbit | Yes | Trauma |
| S05.90 | Unspecified injury of unspecified eye and orbit (open wound of eyeball) | Yes | Trauma |
| T15.0 | Foreign body in cornea, unspecified eye, | Yes | Trauma |
| T15.10 | Foreign body in conjunctival sac, unspecified eye | Yes | Trauma |
| T15.80 | Foreign body in other and multiple parts of external eye, unspecified eye | Yes | Trauma |
| T15.80 | Foreign body in other and multiple parts of external eye, unspecified eye | Yes | Trauma |
| T15.90 | Foreign body on external eye, part unspecified, unspecified eye, | Yes | Trauma |
| T26.00 | Burn of unspecified eyelid and periocular area | Yes | Trauma |
| T26.10 | Burn of cornea and conjunctival sac, unspecified eye | Yes | Trauma |
| T26.20 | Burn with resulting rupture and destruction of unspecified eyeball | Yes | Trauma |
| T26.30 | Burns of other specified parts of unspecified eye and adnexa | Yes | Trauma |
| T26.50 | Corrosion of unspecified eyelid and periocular area | Yes | Trauma |
| T26.60 | Corrosion of cornea and conjunctival sac, unspecified eye | Yes | Trauma |
| T26.60 | Corrosion of cornea and conjunctival sac, unspecified eye | Yes | Trauma |
| Z01.00 | Encounter for examination of eyes and vision without abnormal findings | No | Miscellany |
| Z53.21 | Procedure and treatment not carried out due to patient leaving prior to being seen by health care provider | No | Miscellany |
| H54.20 | Low vision, both eyes | Could not  determine | Miscellany |
