# Supplementary material for: Ocular related emergencies in Spain during the COVID-19 pandemic, a multicenter study
Source: BMC Ophthalmol. 2021 Nov 27;21:408. doi: 10.1186/s12886-021-02169-x (PMC8626758; doi:10.1186/s12886-021-02169-x)
Supplement: Supplementary file 2 — Additional file 2: ETable2. Incidence of diagnosis per year. [file 12886_2021_2169_MOESM2_ESM.docx]

**ETable2. Incidence of diagnosis per year.**

| **ICD‐10 Diagnosis Code and Diagnosis** | | **[ALL]** | **2019** | **2020** |
| --- | --- | --- | --- | --- |
| B00.50 | Herpesviral ocular disease | 82 (0.85%) | 59 (0.76%) | 23 (1.20%) |
| B00.52 | Herpesviral keratitis | 154 (1.60%) | 116 (1.50%) | 38 (1.98%) |
| B60.12 | Conjunctivitis due to Acanthamoeba | 1 (0.01%) | 1 (0.01%) | 0 (0.00%) |
| E08.32 | Diabetes mellitus due to underlying condition with mild nonproliferative diabetic retinopathy | 1 (0.01%) | 1 (0.01%) | 0 (0.00%) |
| E08.34 | Diabetes mellitus due to underlying condition with severe nonproliferative diabetic retinopathy | 1 (0.01%) | 1 (0.01%) | 0 (0.00%) |
| E11.32 | Type 2 diabetes mellitus with mild nonproliferative diabetic retinopathy | 4 (0.04%) | 3 (0.04%) | 1 (0.05%) |
| E13.31 | Other specified diabetes mellitus with unspecified diabetic retinopathy | 2 (0.02%) | 2 (0.03%) | 0 (0.00%) |
| E13.35 | Other specified diabetes mellitus with proliferative diabetic retinopathy | 8 (0.08%) | 7 (0.09%) | 1 (0.05%) |
| G43.1 | Migraine with aura | 71 (0.74%) | 56 (0.72%) | 15 (0.78%) |
| H00.01 | Hordeolum externum | 350 (3.63%) | 287 (3.71%) | 63 (3.28%) |
| H00.023 | Hordeolum internum right eye, unspecified eyelid | 90 (0.93%) | 77 (1.00%) | 13 (0.68%) |
| H00.03 | Abscess of eyelid | 6 (0.06%) | 2 (0.03%) | 4 (0.21%) |
| H00.19 | Chalazion unspecified eye, unspecified eyelid | 32 (0.33%) | 28 (0.36%) | 4 (0.21%) |
| H01.009 | Unspecified blepharitis unspecified eye, unspecified eyelid | 133 (1.38%) | 97 (1.25%) | 36 (1.88%) |
| H01.019 | Ulcerative blepharitis unspecified eye, unspecified eyelid | 1 (0.01%) | 0 (0.00%) | 1 (0.05%) |
| H01.119 | Allergic dermatitis of unspecified eye, unspecified eyelid | 12 (0.12%) | 11 (0.14%) | 1 (0.05%) |
| H01.139 | Eczematous dermatitis of unspecified eye, unspecified eyelid | 14 (0.15%) | 13 (0.17%) | 1 (0.05%) |
| H01.8 | Other specified inflammations of eyelid | 17 (0.18%) | 10 (0.13%) | 7 (0.36%) |
| H01.9 | Unspecified inflammation of eyelid | 39 (0.40%) | 37 (0.48%) | 2 (0.10%) |
| H02.009 | Unspecified entropion of unspecified eye, unspecified eyelid | 11 (0.11%) | 9 (0.12%) | 2 (0.10%) |
| H02.033 | Cicatricial entropion of unspecified eye, unspecified eyelid | 2 (0.02%) | 2 (0.03%) | 0 (0.00%) |
| H02.059 | Trichiasis without entropion unspecified eye, unspecified eyelid | 54 (0.56%) | 45 (0.58%) | 9 (0.47%) |
| H02.109 | Unspecified ectropion of unspecified eye, unspecified eyelid | 9 (0.09%) | 8 (0.10%) | 1 (0.05%) |
| H02.139 | Senile ectropion of unspecified eye, unspecified eyelid | 1 (0.01%) | 1 (0.01%) | 0 (0.00%) |
| H02.239 | Paralytic lagophthalmos unspecified eye, unspecified eyelid | 6 (0.06%) | 2 (0.03%) | 4 (0.21%) |
| H02.409 | Unspecified ptosis of unspecified eyelid | 7 (0.07%) | 1 (0.01%) | 6 (0.31%) |
| H02.519 | Abnormal innervation syndrome unspecified eye, unspecified eyelid | 9 (0.09%) | 8 (0.10%) | 1 (0.05%) |
| H02.539 | Eyelid retraction unspecified eye, unspecified lid | 1 (0.01%) | 1 (0.01%) | 0 (0.00%) |
| H02.70 | Unspecified degenerative disorders of eyelid and periocular area | 2 (0.02%) | 2 (0.03%) | 0 (0.00%) |
| H02.829 | Cysts of unspecified eye, unspecified eyelid | 9 (0.09%) | 9 (0.12%) | 0 (0.00%) |
| H02.839 | Dermatochalasis of unspecified eye, unspecified eyelid | 1 (0.01%) | 1 (0.01%) | 0 (0.00%) |
| H02.849 | Edema of unspecified eye, unspecified eyelid | 60 (0.62%) | 55 (0.71%) | 5 (0.26%) |
| H02.89 | Other specified disorders of eyelid | 9 (0.09%) | 4 (0.05%) | 5 (0.26%) |
| H02.9 | Unspecified disorder of eyelid | 19 (0.20%) | 13 (0.17%) | 6 (0.31%) |
| H04.009 | Unspecified dacryoadenitis, unspecified lacrimal gland | 2 (0.02%) | 1 (0.01%) | 1 (0.05%) |
| H04.019 | Acute dacryoadenitis, unspecified lacrimal gland | 9 (0.09%) | 8 (0.10%) | 1 (0.05%) |
| H04.039 | Chronic enlargement of unspecified lacrimal gland | 1 (0.01%) | 1 (0.01%) | 0 (0.00%) |
| H04.129 | Dry eye syndrome of unspecified lacrimal gland | 239 (2.48%) | 194 (2.51%) | 45 (2.35%) |
| H04.209 | Unspecified epiphora, unspecified side | 3 (0.03%) | 3 (0.04%) | 0 (0.00%) |
| H04.309 | Unspecified dacryocystitis of unspecified lacrimal passage | 3 (0.03%) | 2 (0.03%) | 1 (0.05%) |
| H04.329 | Acute dacryocystitis of unspecified lacrimal passage | 42 (0.44%) | 30 (0.39%) | 12 (0.63%) |
| H04.339 | Acute lacrimal canaliculitis of unspecified lacrimal passage | 2 (0.02%) | 1 (0.01%) | 1 (0.05%) |
| H04.419 | Chronic dacryocystitis of unspecified lacrimal passage | 1 (0.01%) | 1 (0.01%) | 0 (0.00%) |
| H04.539 | Neonatal obstruction of unspecified nasolacrimal duct | 1 (0.01%) | 1 (0.01%) | 0 (0.00%) |
| H04.559 | Acquired stenosis of unspecified nasolacrimal duct | 4 (0.04%) | 4 (0.05%) | 0 (0.00%) |
| H04.569 | Stenosis of unspecified lacrimal punctum | 1 (0.01%) | 1 (0.01%) | 0 (0.00%) |
| H05.00 | Unspecified acute inflammation of orbit | 7 (0.07%) | 3 (0.04%) | 4 (0.21%) |
| H05.01 | Cellulitis of orbit | 3 (0.03%) | 2 (0.03%) | 1 (0.05%) |
| H05.229 | Edema of unspecified orbit | 1 (0.01%) | 1 (0.01%) | 0 (0.00%) |
| H05.239 | Hemorrhage of unspecified orbit | 1 (0.01%) | 1 (0.01%) | 0 (0.00%) |
| H05.339 | Deformity of unspecified orbit due to trauma or surgery | 3 (0.03%) | 3 (0.04%) | 0 (0.00%) |
| H05.89 | Other disorders of orbit | 5 (0.05%) | 5 (0.06%) | 0 (0.00%) |
| H1.009 | Blepharitis | 124 (1.29%) | 88 (1.14%) | 36 (1.88%) |
| H10.019 | Acute follicular conjunctivitis, unspecified eye | 176 (1.82%) | 162 (2.10%) | 14 (0.73%) |
| H10.029 | Other mucopurulent conjunctivitis, unspecified eye | 6 (0.06%) | 6 (0.08%) | 0 (0.00%) |
| H10.10 | Acute atopic conjunctivitis, unspecified eye | 28 (0.29%) | 21 (0.27%) | 7 (0.36%) |
| H10.219 | Acute toxic conjunctivitis, unspecified eye | 25 (0.26%) | 24 (0.31%) | 1 (0.05%) |
| H10.229 | Pseudomembranous conjunctivitis, unspecified eye | 73 (0.76%) | 70 (0.91%) | 3 (0.16%) |
| H10.30 | Unspecified acute conjunctivitis, unspecified eye | 1004 (10.41%) | 896 (11.59%) | 108 (5.63%) |
| H10.409 | Unspecified chronic conjunctivitis, unspecified eye | 5 (0.05%) | 3 (0.04%) | 2 (0.10%) |
| H10.44 | Vernal conjunctivitis | 177 (1.83%) | 139 (1.80%) | 38 (1.98%) |
| H10.45 | Other chronic allergic conjunctivitis | 27 (0.28%) | 27 (0.35%) | 0 (0.00%) |
| H10.45 | Other chronic allergic conjunctivitis | 5 (0.05%) | 1 (0.01%) | 4 (0.21%) |
| H10.539 | Contact blepharoconjunctivitis, unspecified eye | 1 (0.01%) | 0 (0.00%) | 1 (0.05%) |
| H10.819 | Pingueculitis, unspecified eye | 23 (0.24%) | 21 (0.27%) | 2 (0.10%) |
| H10.89 | Other conjunctivitis | 128 (1.33%) | 105 (1.36%) | 23 (1.20%) |
| H10.9 | Unspecified conjunctivitis | 211 (2.19%) | 179 (2.32%) | 32 (1.67%) |
| H11.009 | Unspecified pterygium of unspecified eye | 18 (0.19%) | 16 (0.21%) | 2 (0.10%) |
| H11.10 | Unspecified conjunctival degenerations | 6 (0.06%) | 5 (0.06%) | 1 (0.05%) |
| H11.129 | Conjunctival concretions, unspecified eye | 8 (0.08%) | 6 (0.08%) | 2 (0.10%) |
| H11.139 | Conjunctival pigmentations, unspecified eye | 2 (0.02%) | 1 (0.01%) | 1 (0.05%) |
| H11.159 | Pinguecula, unspecified eye | 13 (0.13%) | 11 (0.14%) | 2 (0.10%) |
| H11.229 | Conjunctival granuloma, unspecified | 4 (0.04%) | 2 (0.03%) | 2 (0.10%) |
| H11.249 | Scarring of conjunctiva, unspecified eye | 1 (0.01%) | 1 (0.01%) | 0 (0.00%) |
| H11.30 | Conjunctival hemorrhage, unspecified eye | 433 (4.49%) | 367 (4.75%) | 66 (3.44%) |
| H11.419 | Vascular abnormalities of conjunctiva, unspecified eye | 1 (0.01%) | 1 (0.01%) | 0 (0.00%) |
| H11.429 | Conjunctival edema, unspecified eye | 1 (0.01%) | 1 (0.01%) | 0 (0.00%) |
| H11.43 | Conjuntival hiperhemia | 81 (0.84%) | 61 (0.79%) | 20 (1.04%) |
| H11.439 | Conjunctival hyperemia, unspecified eye | 2 (0.02%) | 2 (0.03%) | 0 (0.00%) |
| H11.44 | Conjuntival cysts | 6 (0.06%) | 3 (0.04%) | 3 (0.16%) |
| H11.449 | Conjunctival cysts, unspecified eye | 11 (0.11%) | 8 (0.10%) | 3 (0.16%) |
| H11.829 | Conjunctivochalasis, unspecified eye | 2 (0.02%) | 2 (0.03%) | 0 (0.00%) |
| H11.9 | Unspecified disorder of conjunctiva | 1 (0.01%) | 1 (0.01%) | 0 (0.00%) |
| H15.009 | Unspecified scleritis, unspecified eye | 37 (0.38%) | 28 (0.36%) | 9 (0.47%) |
| H15.019 | Anterior scleritis, unspecified eye | 7 (0.07%) | 5 (0.06%) | 2 (0.10%) |
| H15.039 | Posterior scleritis, unspecified eye | 3 (0.03%) | 3 (0.04%) | 0 (0.00%) |
| H15.119 | Episcleritis periodica fugax, unspecified eye | 21 (0.22%) | 20 (0.26%) | 1 (0.05%) |
| H15.129 | Nodular episcleritis, unspecified eye | 11 (0.11%) | 8 (0.10%) | 3 (0.16%) |
| H16.009 | Unspecified corneal ulcer, unspecified eye | 390 (4.04%) | 323 (4.18%) | 67 (3.49%) |
| H16.019 | Central corneal ulcer, unspecified eye | 4 (0.04%) | 4 (0.05%) | 0 (0.00%) |
| H16.049 | Marginal corneal ulcer, unspecified eye | 25 (0.26%) | 23 (0.30%) | 2 (0.10%) |
| H16.079 | Perforated corneal ulcer, unspecified eye | 1 (0.01%) | 1 (0.01%) | 0 (0.00%) |
| H16.109 | Unspecified superficial keratitis, unspecified eye | 34 (0.35%) | 29 (0.38%) | 5 (0.26%) |
| H16.129 | Filamentary keratitis, unspecified eye | 13 (0.13%) | 11 (0.14%) | 2 (0.10%) |
| H16.139 | Photokeratitis, unspecified eye | 3 (0.03%) | 3 (0.04%) | 0 (0.00%) |
| H16.149 | Punctate keratitis, unspecified eye | 546 (5.66%) | 453 (5.86%) | 93 (4.85%) |
| H16.209 | Unspecified keratoconjunctivitis, unspecified eye | 38 (0.39%) | 32 (0.41%) | 6 (0.31%) |
| H16.219 | Exposure keratoconjunctivitis, unspecified eye | 13 (0.13%) | 11 (0.14%) | 2 (0.10%) |
| H16.229 | Keratoconjunctivitis sicca, not specified as Sjogren's, unspecified eye | 26 (0.27%) | 24 (0.31%) | 2 (0.10%) |
| H16.239 | Neurotrophic keratoconjunctivitis, unspecified eye | 1 (0.01%) | 1 (0.01%) | 0 (0.00%) |
| H16.259 | Phlyctenular keratoconjunctivitis, unspecified eye | 5 (0.05%) | 4 (0.05%) | 1 (0.05%) |
| H16.269 | Vernal keratoconjunctivitis, with limbar and corneal involvement, unspecified eye | 2 (0.02%) | 1 (0.01%) | 1 (0.05%) |
| H16.299 | Other keratoconjunctivitis, unspecified eye | 1 (0.01%) | 1 (0.01%) | 0 (0.00%) |
| H16.309 | Unspecified interstitial keratitis, unspecified eye | 5 (0.05%) | 5 (0.06%) | 0 (0.00%) |
| H16.319 | Corneal abscess, unspecified eye | 149 (1.54%) | 118 (1.53%) | 31 (1.62%) |
| H16.339 | Sclerosing keratitis, unspecified eye | 1 (0.01%) | 1 (0.01%) | 0 (0.00%) |
| H16.9 | Unspecified keratitis | 398 (4.13%) | 334 (4.32%) | 64 (3.34%) |
| H17.10 | Central corneal opacity, unspecified eye | 1 (0.01%) | 0 (0.00%) | 1 (0.05%) |
| H17.9 | Unspecified corneal scar and opacity | 24 (0.25%) | 21 (0.27%) | 3 (0.16%) |
| H18.009 | Unspecified corneal deposit, unspecified eye | 1 (0.01%) | 1 (0.01%) | 0 (0.00%) |
| H18.10 | Bullous keratopathy, unspecified eye | 9 (0.09%) | 7 (0.09%) | 2 (0.10%) |
| H18.20 | Unspecified corneal edema | 38 (0.39%) | 30 (0.39%) | 8 (0.42%) |
| H18.239 | Secondary corneal edema, unspecified eye | 3 (0.03%) | 3 (0.04%) | 0 (0.00%) |
| H18.40 | Unspecified corneal degeneration | 1 (0.01%) | 1 (0.01%) | 0 (0.00%) |
| H18.429 | Band keratopathy, unspecified eye | 1 (0.01%) | 0 (0.00%) | 1 (0.05%) |
| H18.51 | Endothelial corneal dystrophy | 2 (0.02%) | 1 (0.01%) | 1 (0.05%) |
| H18.609 | Keratoconus, unspecified, unspecified eye | 3 (0.03%) | 2 (0.03%) | 1 (0.05%) |
| H18.829 | Corneal disorder due to contact lens, unspecified eye | 40 (0.41%) | 33 (0.43%) | 7 (0.36%) |
| H18.839 | Recurrent erosion of cornea, unspecified eye | 45 (0.47%) | 34 (0.44%) | 11 (0.57%) |
| H20.00 | Unspecified acute and subacute iridocyclitis | 292 (3.03%) | 200 (2.59%) | 92 (4.80%) |
| H20.019 | Primary iridocyclitis, unspecified eye | 41 (0.42%) | 31 (0.40%) | 10 (0.52%) |
| H20.029 | Recurrent acute iridocyclitis, unspecified eye | 4 (0.04%) | 3 (0.04%) | 1 (0.05%) |
| H20.039 | Secondary infectious iridocyclitis, unspecified eye | 14 (0.15%) | 11 (0.14%) | 3 (0.16%) |
| H20.049 | Secondary noninfectious iridocyclitis, unspecified eye | 33 (0.34%) | 21 (0.27%) | 12 (0.63%) |
| H20.10 | Chronic iridocyclitis, unspecified eye | 7 (0.07%) | 7 (0.09%) | 0 (0.00%) |
| H20.20 | Lens-induced iridocyclitis, unspecified eye | 3 (0.03%) | 3 (0.04%) | 0 (0.00%) |
| H20.819 | Fuchs' heterochromic cyclitis, unspecified eye | 4 (0.04%) | 3 (0.04%) | 1 (0.05%) |
| H20.9 | Unspecified iridocyclitis | 6 (0.06%) | 4 (0.05%) | 2 (0.10%) |
| H21.00 | Hyphema, unspecified eye | 16 (0.17%) | 8 (0.10%) | 8 (0.42%) |
| H21.1X9 | Other vascular disorders of iris and ciliary body, unspecified eye | 3 (0.03%) | 3 (0.04%) | 0 (0.00%) |
| H21.40 | Pupillary membranes, unspecified eye | 2 (0.02%) | 1 (0.01%) | 1 (0.05%) |
| H21.539 | Iridodialysis, unspecified eye | 1 (0.01%) | 1 (0.01%) | 0 (0.00%) |
| H21.569 | Pupillary abnormality, unspecified eye | 1 (0.01%) | 1 (0.01%) | 0 (0.00%) |
| H25.019 | Cortical age-related cataract, unspecified eye | 2 (0.02%) | 1 (0.01%) | 1 (0.05%) |
| H25.049 | Posterior subcapsular polar age-related cataract, unspecified eye | 6 (0.06%) | 5 (0.06%) | 1 (0.05%) |
| H25.89 | Other age-related cataract | 1 (0.01%) | 1 (0.01%) | 0 (0.00%) |
| H25.9 | Unspecified age-related cataract | 17 (0.18%) | 8 (0.10%) | 9 (0.47%) |
| H26.009 | Unspecified infantile and juvenile cataract, unspecified eye | 6 (0.06%) | 0 (0.00%) | 6 (0.31%) |
| H26.8 | Other specified cataract | 8 (0.08%) | 5 (0.06%) | 3 (0.16%) |
| H26.9 | Unspecified cataract | 68 (0.70%) | 60 (0.78%) | 8 (0.42%) |
| H27.119 | Subluxation of lens, unspecified eye | 8 (0.08%) | 5 (0.06%) | 3 (0.16%) |
| H27.8 | Other specified disorders of lens | 1 (0.01%) | 1 (0.01%) | 0 (0.00%) |
| H30.009 | Unspecified focal chorioretinal inflammation, unspecified eye | 22 (0.23%) | 20 (0.26%) | 2 (0.10%) |
| H30.819 | Harada's disease, unspecified eye | 2 (0.02%) | 1 (0.01%) | 1 (0.05%) |
| H30.90 | Unspecified chorioretinal inflammation, unspecified eye | 2 (0.02%) | 0 (0.00%) | 2 (0.10%) |
| H31.019 | Unspecified chorioretinal scars, unspecified eye | 3 (0.03%) | 3 (0.04%) | 0 (0.00%) |
| H31.129 | Macula scars of posterior pole (postinflammatory) (post-traumatic), unspecified eye | 1 (0.01%) | 1 (0.01%) | 0 (0.00%) |
| H31.429 | Serous choroidal detachment, unspecified eye | 1 (0.01%) | 1 (0.01%) | 0 (0.00%) |
| H31.8 | Other specified disorders of choroid | 3 (0.03%) | 3 (0.04%) | 0 (0.00%) |
| H33.00 | Unspecified retinal detachment with retinal break | 33 (0.34%) | 25 (0.32%) | 8 (0.42%) |
| H33.00Ê | Unspecified retinal detachment with retinal break, unspecified eye | 28 (0.29%) | 16 (0.21%) | 12 (0.63%) |
| H33.019 | Retinal detachment with single break, unspecified eye | 8 (0.08%) | 5 (0.06%) | 3 (0.16%) |
| H33.029 | Retinal detachment with multiple breaks, unspecified eye | 1 (0.01%) | 0 (0.00%) | 1 (0.05%) |
| H33.059 | Total retinal detachment, unspecified eye | 3 (0.03%) | 1 (0.01%) | 2 (0.10%) |
| H33.109 | Unspecified retinoschisis, unspecified eye | 3 (0.03%) | 2 (0.03%) | 1 (0.05%) |
| H33.309 | Unspecified retinal break, unspecified eye | 51 (0.53%) | 33 (0.43%) | 18 (0.94%) |
| H33.319 | Horseshoe tear of retina without detachment, unspecified eye | 36 (0.37%) | 30 (0.39%) | 6 (0.31%) |
| H33.329 | Round hole, unspecified eye | 1 (0.01%) | 1 (0.01%) | 0 (0.00%) |
| H33.40 | Traction detachment of retina, unspecified eye | 2 (0.02%) | 2 (0.03%) | 0 (0.00%) |
| H34.00 | Transient retinal artery occlusion, unspecified eye | 15 (0.16%) | 11 (0.14%) | 4 (0.21%) |
| H34.10 | Central retinal artery occlusion, unspecified eye | 1 (0.01%) | 1 (0.01%) | 0 (0.00%) |
| H34.239 | Retinal artery branch occlusion, unspecified eye | 3 (0.03%) | 3 (0.04%) | 0 (0.00%) |
| H34.81 | Central retinal vein occlusion | 9 (0.09%) | 7 (0.09%) | 2 (0.10%) |
| H34.829 | Venous engorgement, unspecified eye | 5 (0.05%) | 5 (0.06%) | 0 (0.00%) |
| H34.83 | Tributary (branch) retinal vein occlusion | 23 (0.24%) | 11 (0.14%) | 12 (0.63%) |
| H35.039 | Hypertensive retinopathy, unspecified eye | 2 (0.02%) | 0 (0.00%) | 2 (0.10%) |
| H35.059 | Retinal neovascularization, unspecified, unspecified eye | 20 (0.21%) | 13 (0.17%) | 7 (0.36%) |
| H35.30 | Unspecified macular degeneration | 20 (0.21%) | 18 (0.23%) | 2 (0.10%) |
| H35.3190 | Nonexudative age-related macular degeneration, unspecified eye, stage unspecified | 35 (0.36%) | 28 (0.36%) | 7 (0.36%) |
| H35.32 | Exudative age-related macular degeneration | 47 (0.49%) | 31 (0.40%) | 16 (0.83%) |
| H35.349 | Macular cyst, hole, or pseudohole, unspecified eye | 9 (0.09%) | 6 (0.08%) | 3 (0.16%) |
| H35.359 | Cystoid macular degeneration, unspecified eye | 19 (0.20%) | 15 (0.19%) | 4 (0.21%) |
| H35.379 | Puckering of macula, unspecified eye | 2 (0.02%) | 2 (0.03%) | 0 (0.00%) |
| H35.389 | Toxic maculopathy, unspecified eye | 1 (0.01%) | 1 (0.01%) | 0 (0.00%) |
| H35.40 | Unspecified peripheral retinal degeneration | 1 (0.01%) | 1 (0.01%) | 0 (0.00%) |
| H35.419 | Lattice degeneration of retina, unspecified eye | 4 (0.04%) | 0 (0.00%) | 4 (0.21%) |
| H35.60 | Retinal hemorrhage, unspecified eye | 16 (0.17%) | 11 (0.14%) | 5 (0.26%) |
| H35.719 | Central serous chorioretinopathy, unspecified eye | 12 (0.12%) | 9 (0.12%) | 3 (0.16%) |
| H35.729 | Serous detachment of retinal pigment epithelium, unspecified eye | 1 (0.01%) | 0 (0.00%) | 1 (0.05%) |
| H35.81 | Retinal edema | 5 (0.05%) | 1 (0.01%) | 4 (0.21%) |
| H35.82 | Retinal ischemia | 1 (0.01%) | 1 (0.01%) | 0 (0.00%) |
| H35.89 | Other specified retinal disorders | 3 (0.03%) | 3 (0.04%) | 0 (0.00%) |
| H35.89 | Other specified retinal disorders | 16 (0.17%) | 15 (0.19%) | 1 (0.05%) |
| H35.9 | Unspecified retinal disorder | 8 (0.08%) | 6 (0.08%) | 2 (0.10%) |
| H40.009 | Preglaucoma, unspecified, unspecified eye | 6 (0.06%) | 3 (0.04%) | 3 (0.16%) |
| H40.049 | Steroid responder, unspecified eye | 1 (0.01%) | 1 (0.01%) | 0 (0.00%) |
| H40.059 | Ocular hypertension, unspecified eye | 1 (0.01%) | 1 (0.01%) | 0 (0.00%) |
| H40.059 | Ocular hypertension, unspecified eye | 51 (0.53%) | 41 (0.53%) | 10 (0.52%) |
| H40.10 | Unspecified open-angle glaucoma | 18 (0.19%) | 15 (0.19%) | 3 (0.16%) |
| H40.1194 | Primary open-angle glaucoma, unspecified eye, indeterminate stage | 6 (0.06%) | 5 (0.06%) | 1 (0.05%) |
| H40.1490 | Capsular glaucoma with pseudoexfoliation of lens, unspecified eye, stage unspecified | 1 (0.01%) | 1 (0.01%) | 0 (0.00%) |
| H40.20 | Unspecified primary angle-closure glaucoma | 1 (0.01%) | 1 (0.01%) | 0 (0.00%) |
| H40.219 | Acute angle-closure glaucoma, unspecified eye | 26 (0.27%) | 25 (0.32%) | 1 (0.05%) |
| H40.239 | Intermittent angle-closure glaucoma, unspecified eye | 1 (0.01%) | 0 (0.00%) | 1 (0.05%) |
| H40.40 | Glaucoma secondary to eye inflammation, unspecified eye | 1 (0.01%) | 0 (0.00%) | 1 (0.05%) |
| H40.40X4 | Glaucoma secondary to eye inflammation, unspecified eye, indeterminate stage | 2 (0.02%) | 0 (0.00%) | 2 (0.10%) |
| H40.50 | Glaucoma secondary to other eye disorders, unspecified eye | 9 (0.09%) | 5 (0.06%) | 4 (0.21%) |
| H40.89 | Other specified glaucoma | 6 (0.06%) | 3 (0.04%) | 3 (0.16%) |
| H40.9 | Unspecified glaucoma | 9 (0.09%) | 7 (0.09%) | 2 (0.10%) |
| H43.10 | Vitreous hemorrhage, unspecified eye | 52 (0.54%) | 28 (0.36%) | 24 (1.25%) |
| H43.399 | Other vitreous opacities, unspecified eye | 6 (0.06%) | 6 (0.08%) | 0 (0.00%) |
| H43.819 | Vitreous degeneration, unspecified eye | 678 (7.03%) | 500 (6.47%) | 178 (9.28%) |
| H43.9 | Unspecified disorder of vitreous body | 2 (0.02%) | 2 (0.03%) | 0 (0.00%) |
| H44.009 | Unspecified purulent endophthalmitis, unspecified eye | 39 (0.40%) | 24 (0.31%) | 15 (0.78%) |
| H44.119 | Panuveitis, unspecified eye | 9 (0.09%) | 8 (0.10%) | 1 (0.05%) |
| H44.20 | Degenerative myopia, unspecified eye | 22 (0.23%) | 15 (0.19%) | 7 (0.36%) |
| H44.40 | Unspecified hypotony of eye | 2 (0.02%) | 2 (0.03%) | 0 (0.00%) |
| H44.419 | Flat anterior chamber hypotony of unspecified eye | 1 (0.01%) | 1 (0.01%) | 0 (0.00%) |
| H44.429 | Hypotony of unspecified eye due to ocular fistula | 1 (0.01%) | 0 (0.00%) | 1 (0.05%) |
| H44.519 | Absolute glaucoma, unspecified eye | 4 (0.04%) | 4 (0.05%) | 0 (0.00%) |
| H44.529 | Atrophy of globe, unspecified eye | 2 (0.02%) | 0 (0.00%) | 2 (0.10%) |
| H44.719 | Retained (nonmagnetic) (old) foreign body in anterior chamber, unspecified eye | 4 (0.04%) | 2 (0.03%) | 2 (0.10%) |
| H46.8 | Other optic neuritis | 1 (0.01%) | 1 (0.01%) | 0 (0.00%) |
| H46.9 | Unspecified optic neuritis | 3 (0.03%) | 0 (0.00%) | 3 (0.16%) |
| H47.019 | Ischemic optic neuropathy, unspecified eye | 1 (0.01%) | 1 (0.01%) | 0 (0.00%) |
| H47.019 | Ischemic optic neuropathy, unspecified eye | 14 (0.15%) | 7 (0.09%) | 7 (0.36%) |
| H47.029 | Hemorrhage in optic nerve sheath, unspecified eye | 1 (0.01%) | 1 (0.01%) | 0 (0.00%) |
| H47.099 | Other disorders of optic nerve, not elsewhere classified, unspecified eye | 1 (0.01%) | 1 (0.01%) | 0 (0.00%) |
| H47.11 | Papilledema associated with increased intracranial pressure | 1 (0.01%) | 1 (0.01%) | 0 (0.00%) |
| H47.239 | Glaucomatous optic atrophy, unspecified eye | 1 (0.01%) | 1 (0.01%) | 0 (0.00%) |
| H47.299 | Other optic atrophy, unspecified eye | 1 (0.01%) | 0 (0.00%) | 1 (0.05%) |
| H47.329 | Drusen of optic disc, unspecified eye | 1 (0.01%) | 1 (0.01%) | 0 (0.00%) |
| H47.9 | Unspecified disorder of visual pathways | 1 (0.01%) | 0 (0.00%) | 1 (0.05%) |
| H49.00 | Third [oculomotor] nerve palsy, unspecified eye | 2 (0.02%) | 0 (0.00%) | 2 (0.10%) |
| H49.10 | Fourth [trochlear] nerve palsy, unspecified eye | 14 (0.15%) | 8 (0.10%) | 6 (0.31%) |
| H49.20 | Sixth [abducent] nerve palsy, unspecified eye | 12 (0.12%) | 7 (0.09%) | 5 (0.26%) |
| H49.30 | Total (external) ophthalmoplegia, unspecified eye | 1 (0.01%) | 0 (0.00%) | 1 (0.05%) |
| H50.00 | Unspecified esotropia | 2 (0.02%) | 1 (0.01%) | 1 (0.05%) |
| H50.10 | Unspecified exotropia | 1 (0.01%) | 1 (0.01%) | 0 (0.00%) |
| H50.51 | Esophoria | 1 (0.01%) | 1 (0.01%) | 0 (0.00%) |
| H51.20 | Internuclear ophthalmoplegia, unspecified eye | 1 (0.01%) | 0 (0.00%) | 1 (0.05%) |
| H52.10 | Myopia, unspecified eye | 7 (0.07%) | 7 (0.09%) | 0 (0.00%) |
| H52.209 | Unspecified astigmatism, unspecified eye | 1 (0.01%) | 1 (0.01%) | 0 (0.00%) |
| H52.6 | Other disorders of refraction | 11 (0.11%) | 5 (0.06%) | 6 (0.31%) |
| H52.6 | Other disorders of refraction | 1 (0.01%) | 0 (0.00%) | 1 (0.05%) |
| H52.7 | Unspecified disorder of refraction | 6 (0.06%) | 4 (0.05%) | 2 (0.10%) |
| H53.149 | Visual discomfort, unspecified | 83 (0.86%) | 56 (0.72%) | 27 (1.41%) |
| H53.19 | Other subjective visual disturbances | 1 (0.01%) | 0 (0.00%) | 1 (0.05%) |
| H53.2 | Diplopia | 63 (0.65%) | 46 (0.60%) | 17 (0.89%) |
| H53.419 | Scotoma involving central area, unspecified eye | 1 (0.01%) | 1 (0.01%) | 0 (0.00%) |
| H53.8 | Other visual disturbances | 101 (1.05%) | 68 (0.88%) | 33 (1.72%) |
| H53.9 | Unspecified visual disturbance | 27 (0.28%) | 24 (0.31%) | 3 (0.16%) |
| H54.2X | Low vision, both eyes, different category levels | 2 (0.02%) | 2 (0.03%) | 0 (0.00%) |
| H54.50 | Low vision, one eye, unspecified eye | 2 (0.02%) | 2 (0.03%) | 0 (0.00%) |
| H54.60 | Unqualified visual loss, one eye, unspecified | 10 (0.10%) | 8 (0.10%) | 2 (0.10%) |
| H57.02 | Anisocoria | 6 (0.06%) | 4 (0.05%) | 2 (0.10%) |
| H57.04 | Mydriasis | 6 (0.06%) | 3 (0.04%) | 3 (0.16%) |
| H57.059 | Tonic pupil, unspecified eye | 3 (0.03%) | 3 (0.04%) | 0 (0.00%) |
| H57.10 | Ocular pain, unspecified eye | 35 (0.36%) | 31 (0.40%) | 4 (0.21%) |
| H59.02 | Cataract (lens) fragments in the eye after cataract surgery | 4 (0.04%) | 2 (0.03%) | 2 (0.10%) |
| S00.10 | Contusion of unspecified eyelid and periocular area | 40 (0.41%) | 30 (0.39%) | 10 (0.52%) |
| S00.2 | Other and unspecified superficial injuries of eyelid and periocular area | 7 (0.07%) | 5 (0.06%) | 2 (0.10%) |
| S01.109 | Unspecified open wound of unspecified eyelid and periocular area | 18 (0.19%) | 17 (0.22%) | 1 (0.05%) |
| S01.111 | Laceration without foreign body of right eyelid and periocular area, | 6 (0.06%) | 5 (0.06%) | 1 (0.05%) |
| S02.3 | Fracture of orbital floor | 5 (0.05%) | 5 (0.06%) | 0 (0.00%) |
| S04.019 | Injury of optic nerve, unspecified eye | 1 (0.01%) | 1 (0.01%) | 0 (0.00%) |
| S05.0 | Injury of conjunctiva and corneal abrasion without foreign body, unspecified eye | 117 (1.21%) | 105 (1.36%) | 12 (0.63%) |
| S05.00 | Injury of conjunctiva and corneal abrasion without foreign body, unspecified eye | 10 (0.10%) | 10 (0.13%) | 0 (0.00%) |
| S05.00 | Contusion of eyeball and orbital tissues, unspecified eye | 138 (1.43%) | 110 (1.42%) | 28 (1.46%) |
| S05.10 | Contusion of eyeball and orbital tissues, unspecified eye | 12 (0.12%) | 10 (0.13%) | 2 (0.10%) |
| S05.30 | Ocular laceration without prolapse or loss of intraocular tissue, unspecified eye | 3 (0.03%) | 3 (0.04%) | 0 (0.00%) |
| S05.40 | Penetrating wound of orbit with or without foreign body | 2 (0.02%) | 1 (0.01%) | 1 (0.05%) |
| S05.50 | Penetrating wound with foreign body of unspecified eyeball | 1 (0.01%) | 0 (0.00%) | 1 (0.05%) |
| S05.60 | Penetrating wound without foreign body of unspecified eyeball | 12 (0.12%) | 9 (0.12%) | 3 (0.16%) |
| S05.90 | Unspecified injury of unspecified eye and orbit, | 30 (0.31%) | 16 (0.21%) | 14 (0.73%) |
| S5.00 | Injury of conjunctiva and corneal abrasion without foreign body, unspecified eye | 6 (0.06%) | 6 (0.08%) | 0 (0.00%) |
| T15.0 | Foreign body in cornea, unspecified eye, | 286 (2.96%) | 225 (2.91%) | 61 (3.18%) |
| T15.10 | Foreign body in conjunctival sac, unspecified eye | 60 (0.62%) | 51 (0.66%) | 9 (0.47%) |
| T15.80 | Foreign body in other and multiple parts of external eye, unspecified eye | 41 (0.42%) | 39 (0.50%) | 2 (0.10%) |
| T15.90 | Foreign body on external eye, part unspecified, unspecified eye, | 1 (0.01%) | 1 (0.01%) | 0 (0.00%) |
| T26.50 | Corrosion of unspecified eyelid and periocular area | 1 (0.01%) | 1 (0.01%) | 0 (0.00%) |
| T26.60 | Corrosion of cornea and conjunctival sac, unspecified eye | 14 (0.15%) | 11 (0.14%) | 3 (0.16%) |
| T86.840 | Corneal transplant rejection | 3 (0.03%) | 0 (0.00%) | 3 (0.16%) |
| Z01.00 | Encounter for examination of eyes and vision without abnormal findings | 299 (3.10%) | 241 (3.12%) | 58 (3.02%) |
| Z53.21 | Procedure and treatment not carried out due to patient leaving prior to being seen by health care provider | 71 (0.74%) | 66 (0.85%) | 5 (0.26%) |
|  |  |  |  |  |
|  |  |  |  |  |
